# Cytidine deaminase deficiency in tumor cells is associated with sensitivity to a naphthol derivative and a decrease in oncometabolite levels

**DOI:** 10.1007/s00018-022-04487-9

**Published:** 2022-08-04

**Authors:** Hamza Mameri, Géraldine Buhagiar-Labarchède, Gaëlle Fontaine, Céline Corcelle, Caroline Barette, Rosine Onclercq-Delic, Claire Beauvineau, Florence Mahuteau-Betzer, Mounira Amor-Guéret

**Affiliations:** 1 Institut Curie, PSL Research University, CNRS UMR 3348, 91405 Orsay, France; 2 CNRS UMR 3348, Centre Universitaire, Bât. 110. 91405, Orsay, France; 3 Université Paris-Saclay, CNRS UMR 3348, 91405 Orsay, France; 4 Institut Curie, PSL Research University, CNRS UMR 9187, INSERM U1196, 91405 Orsay, France; 5 CNRS UMR 9187, INSERM, U1196, Centre Universitaire, Bât. 110, 91405 Orsay, France; 6 Université Paris-Saclay, CNRS UMR 9187, INSERM U1196, 91405 Orsay, France; 7 CEA/IRIG/Gen & Chem, Univ. Grenoble Alpes, 38000 Grenoble, France; 8 Present Address: Present address: UMR 1208 IATE, Montpellier University, INRAE, Institut Agro, 34060 Montpellier, France

**Keywords:** Cytidine deaminase, Cell metabolism, Drug sensitivity, MAPT, Cancer therapy

## Abstract

Identifying new molecular targets for novel anticancer treatments is a major challenge in clinical cancer research. We have shown that cytidine deaminase (CDA) expression is downregulated in about 60% of cancer cells and tissues. In this study, we aimed to develop a new anticancer treatment specifically inhibiting the growth of CDA-deficient tumor cells. High-throughput screening of a chemical library led to the identification of a naphthol derivative, X55, targeting CDA-deficient tumor cells preferentially, without affecting the growth of non-tumoral cells regardless of CDA expression status. Metabolomic profiling revealed that CDA-deficient HeLa cells differed markedly from control HeLa cells. X55 treatment had a moderate effect on control cells, but greatly disturbed the metabolome of CDA-deficient HeLa cells, worsening the deregulation of many metabolites. In particular, the levels of the three oncometabolites, fumarate, succinate and 2-hydroxyglutarate, were significantly lower in CDA-depleted cells, and this decrease in levels was exacerbated by X55 treatment, revealing an unexpected link between CDA deficiency, mitochondrial function and X55 response. Finally, we identified strong downregulation of *MAPT* (encoding Tau, a microtubule associated protein) expression as a reliable predictive marker for tumor cell X55 sensitivity.

**Supplementary Information:**

The online version contains supplementary material available at 10.1007/s00018-022-04487-9.

## Introduction

Despite major advances in the development of chemotherapy, many cancers continue to have a poor prognosis, due to the resistance of cancer cells to antineoplastic drugs through intrinsic or acquired mechanisms [1]. Several studies have revealed the major role of alterations to metabolic pathways in cancer initiation and progression, and in resistance to anticancer therapies [2–4]. In particular, some events, such as mitochondrial dysfunction and hypoxia, have been reported to lead to an aberrant accumulation of oncometabolites that drives tumorigenesis; the production pathways for these oncometabolites constitute possible new treatment targets [2–6].

Cytidine deaminase (CDA) is a key enzyme in the production of pyrimidine nucleotide metabolites through the salvage pathway, which catalyzes the hydrolytic deamination of cytidine and deoxycytidine to uridine and deoxyuridine, respectively [7]. We previously reported that the pyrimidine pool disequilibrium resulting from CDA deficiency leads to a decrease in the basal activity of poly(ADP-ribose) polymerase 1 (PARP-1), a multifunctional enzyme involved in many cellular processes, thereby contributing to the generation of genetic instability in CDA-deficient cells [8–11]. We also found that Tau protein is important for the survival of CDA-deficient cells [12], and that CDA expression is downregulated in about 50–88% of cancer cells and tissues of different origins [13]. CDA deficiency, thus, identifies a new subgroup of cancers and appears to be a novel and relevant predictive marker of susceptibility to antitumor drugs, opening up new possibilities for treating cancer [13].

CDA expression levels in tumors have been considered as a potential target for anticancer treatment only in the context of overexpression [14, 15]. However, our results suggest that CDA deficiency could also be used to identify new drugs specifically targeting CDA-deficient tumors and sparing healthy tissues.

In this study, we screened the Institut Curie-CNRS chemical library and identified X55, a naphthol derivative, which targeted 40% of CDA-deficient tumor cell lines and 22% of CDA-proficient tumor cell lines, and had no effect on the growth of non-tumoral cells, regardless of their CDA expression status. We also analyzed and compared the metabolomes of CDA-deficient and proficient cells, with and without X55 treatment. We found that the levels of 228 metabolites were deregulated in CDA-deficient cells relative to control cells. The metabolites concerned included several TCA (tricarboxylic) cycle metabolites. X55 treatment greatly aggravated the deregulation of the levels of many metabolites in these cells, worsening the decreases in the levels of three oncometabolites—succinate, fumarate and 2-hydroxyglutarate—in particular. Finally, based on our previous work identifying Tau protein as crucial to the survival of CDA-deficient cells [12], we checked the levels of *MAPT* expression in X55-treated cells. We found that X55 treatment induced a significant downregulation of *MAPT* expression in X55-sensitive cell lines, but not in X55-resistant cell lines, regardless of CDA expression status. A decrease in *MAPT* expression therefore appears to be a predictive marker of tumor cell sensitivity to X55.

## Results

### Identification of a molecule, X55, preferentially targeting CDA-deficient tumor cells

We screened the CNRS-Institut Curie chemical library (8560 compounds) to identify new anticancer drugs specifically targeting CDA deficiency versus proficiency in tumor cells. We screened the compounds for their ability to affect cell viability differentially, using an isogenic cell model of CDA deficiency: a HeLa cell line stably expressing an adenoviral short hairpin RNA (shRNA) specific for CDA, which therefore displays strong CDA downregulation (HeLa-shCDA) and its control counterpart expressing endogenous CDA (HeLa-Ctrl) [8].

**Fig. 1:**
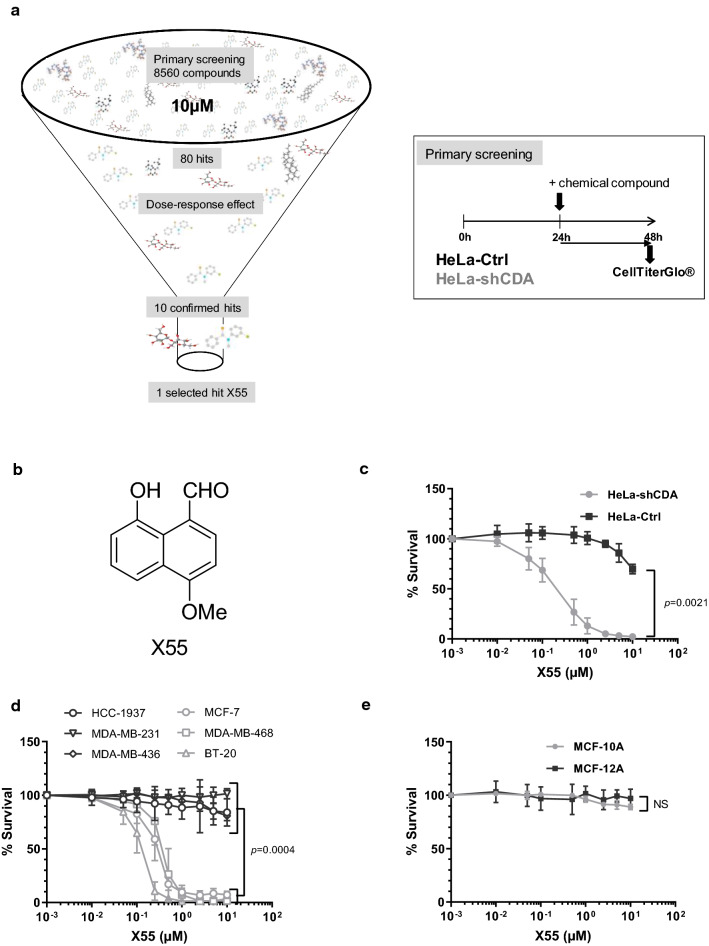
Identification of a naphthol derivative, X55, preferentially targeting CDA-deficient tumor cells. **a** High-throughput screening of the Institut Curie-CNRS Chemical Library. **b** Structure of X55. **c** Isogenic HeLa cell lines (black, control HeLa cells; gray, CDA-depleted HeLa cells) were treated for 72 h with the indicated concentrations of X55, and the percentage of cells surviving is shown. **d** Breast cancer cell lines (black, CDA-proficient cells; gray, CDA-deficient cells) were treated for 72 h with the indicated concentrations of X55, and the percentage of cells surviving is shown. **e** Nonmalignant breast cell lines (black, CDA-proficient cells; gray, CDA-deficient cells) were treated for 72 h with the indicated concentrations of X55, and the percentage of cells surviving is shown. For (**c**) (**d**) and (**e**), cell viability was assessed in MTT assays. The *p* values were calculated in paired *t* tests (**c**) or unpaired *t* tests (**d**, **e**). The error bars represent the means ± SD for three to ten independent experiments (except for MDA-MB-436, 2 independent experiments). *P* < 0.05 was considered statistically significant

In the primary screen, 80 compounds were selected on their ability to induce, at a final concentration of 10 µM, a stronger decrease in viability in CDA-deficient cells than in their CDA-proficient counterparts, with a differential activity of 27.7–99.6% (Fig. 1a). These 80 primary hit compounds were tested again, in a differential dose–effect secondary screen. Ten secondary hits were confirmed to induce lower cell viability in CDA-deficient than in CDA-proficient cells, with a differential effect of 3–32%, at a final concentration of 10 µM. One of these hits, a naphthol derivative, X55 (8-hydroxy-4-methyoxy-1-naphthalene-carboxaldehyde), was finally selected for further biological characterization (Fig. 1b). X55 strongly decreased the viability of CDA-deficient cells at concentrations ranging from 0.2 to 10 μM, but had no major effect on CDA-proficient cells (Fig. 1c).

We then tested the cytotoxicity of X55 on 29 tumor cell lines, nine of which expressed CDA (the other 20 having no CDA expression), and on five nonmalignant cell lines (Table 1). The CDA expression status of all the cell lines was checked (Table 1 and Supplementary Fig. 1). We found that X55 inhibited the growth of 40% of CDA-deficient tumor cell lines (8 of 20) and 22% of CDA-proficient tumor cell lines (2 of 9), without affecting the growth of non-tumor cell lines, regardless of CDA expression status (Fig. 1d and e and Table 1). The sensitivity of cells to X55 treatment was then established from the percent survival of HeLa control cells (70%) and CDA-depleted HeLa cells (2%) incubated with 10 μM X55 for 72 h. Cells were considered highly sensitive if 1 to 10% of cells survived in the presence of 10 μM X55, sensitive if 11–50% of cells survived in these conditions and resistant otherwise (more than 50% of cells surviving in the presence of 10 μM X55) (Table 1).

**Table 1:** List of the cell lines tested for X55 sensitivity

Cell line	CDA status	% survival 10 µM	Response to X55
Cervical cancer			
HeLa-Ctrl	+	70	R
HeLa-shCDA	−	2	HS
Breast cancer			
MCF-7	−	7	HS
BT-20	−	2	HS
MDA-MB-468	−	2	HS
ZR75-1	−	5	HS
T47D	−	34	S
HCC-1428	−	85	R
HCC-1187	−	57	R
HCC-38	−	76	R
Hs578T	−	77	R
HCC-1954	−	83	R
BT-474	−	64	R
BT-549	−	76	R
HCC-1143	+	9	HS
HCC-70	+	27	S
MDA-MB-436	+	85	R
MDA-MB-231	+	101	R
HCC-1937	+	84	R
Melanoma			
A2058	−	44	S
A375	−	93	R
MEL888	−	77	R
MEL624	−	73	R
Lung cancer			
H23	−	86	R
H522	−	85	R
HOP-92	+	90	R
HOP-62	+	80	R
Ovarian cancer			
IGROV-1	−	50	S
SKOV-3	+	101	R
Nonmalignant cell lines			
MRC-5	−	102	R
HEK293T	−	87	R
GM08505	−	86	R
MCF-10A	−	89	R
MCF-12A	+	97	R

*CDA* mRNA levels were presented by Mameri et al. [13] for HeLa-Ctrl, HeLashCDA, ZR75-1, T47D, HCC-1428, BT-474, MCF-7, MDA-MB-468, MDA-MB-231, MDA-MB-436, HCC-38, HCC-70, HCC-1187, HCC-1937, HCC-1143, BT-20, BT-549, HCC-1954, Hs578T, GM8505B, H522, H23, HOP-92, HOP-62, IGROV-1 and SKOV-3 cells. *CDA* mRNA levels for MCF-10A, MCF-12A, MEL888, MEL624, MRC-5, HEK-293T, A2058 and A375 cells are presented in Supplementary Fig. 1

*HS* highly sensitive, *S* sensitive, *R* resistant, (−) CDA-deficient cell line, ( +) CDA-proficient cell line

Finally, to determine whether the sensitivity of CDA-deficient cells was due to the loss of CDA activity, we treated the CDA-expressing HeLa-Ctrl cells with tetrahydrouridine (THU), a well-known CDA inhibitor [16]. The treatment of HeLa control cells with 100 μM THU resulted in a significant increase in the frequency of ultrafine anaphase bridges (UFBs), as previously reported [8] (Supplementary Fig. 2a), confirming the inhibition of CDA activity in these cells. The cells were then treated with 10 μM X55. The inhibition of CDA activity by THU did not sensitize cells to X55 (Supplementary Fig. 2b), suggesting that CDA activity is dispensable for X55 cytotoxicity.

We found that X55 preferentially targeted CDA-deficient tumor cells, although two CDA-expressing tumor cell lines were also sensitive, and that X55 had no effect on the growth of nonmalignant cells, regardless of their CDA expression status, or on CDA-expressing HeLa-Ctrl cells in which CDA activity had been inhibited.

### X55 treatment accentuates the deregulation of several metabolomic pathways in CDA-depleted cells

**Fig. 2:**
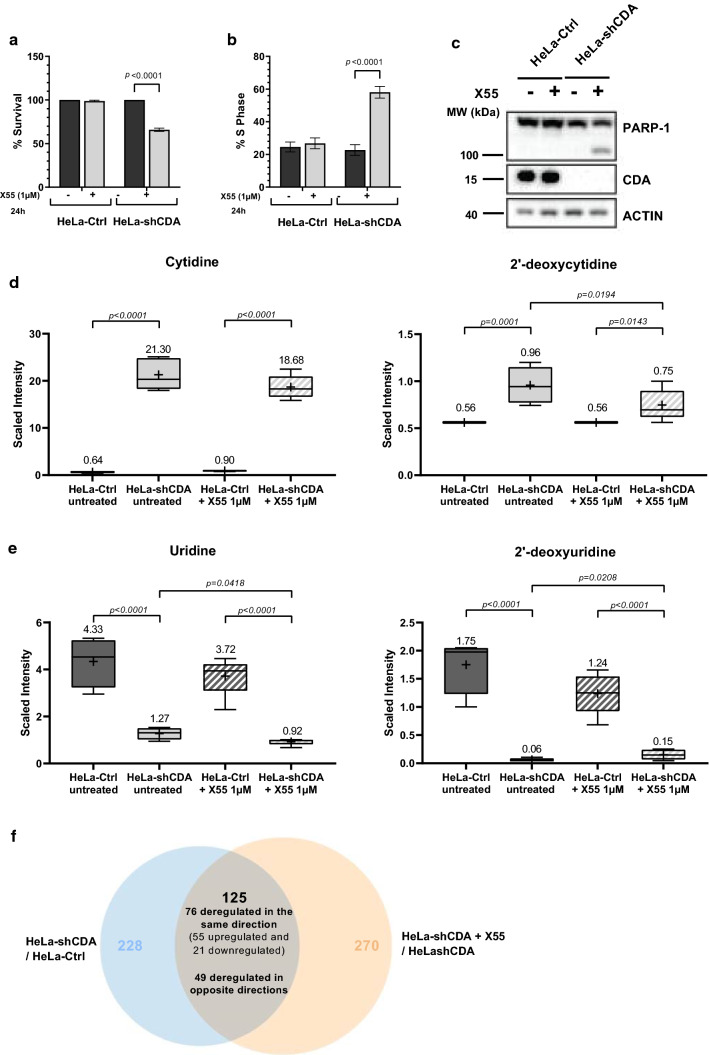
X55 treatment significantly disturbed the cell cycle and metabolome in CDA-depleted HeLa cells. **a** Control HeLa cells (HeLa-Ctrl) and CDA-depleted HeLa cells (HeLa-shCDA) were left untreated (black bars) or were treated for 24 h with 1 μM X55 (gray bars), and the percentage of cells surviving is shown. **b** Percentage of HeLa-Ctrl cells and CDA-depleted HeLa cells in S phase after being left untreated (black bars) or treated with 1 μM X55 for 24 h (gray bars). For (**a**) and (**b**), the error bars represent means ± SD for five independent experiments. The significance of differences was assessed by two-way ANOVA. *P* < 0.05 was considered statistically significant. **c** Amounts of PARP-1 and CDA, assessed by immunoblotting, in the cell lines indicated. Actin was used as a loading control. **d** Measurement of the levels of cytidine (left panel) and 2’-deoxycytidine (right panel) in HeLa-Ctrl cells (dark gray) and CDA-depleted HeLa cells (light gray) left untreated (block-shaded) or treated for 24 h with 1 μM X55 (hatched). **e** Measurement of the levels of uridine (left panel) and 2’-deoxyuridine (right panel) in HeLa-Ctrl cells (dark gray) and CDA-depleted HeLa cells (light gray) left untreated (block-shaded) or treated for 24 h with 1 μM X55 (hatched). For (**d**) and (**e**), the error bars represent means ± SD for four or five independent experiments. The significance of differences was assessed by two-way ANOVA. *P* < 0.05 was considered statistically significant. **f** Venn diagram showing the number of metabolites identified as differentially expressed in HeLa-shCDA cells and HeLa-Ctrl cells (228, blue circle) or in HeLa-shCDA cells with and without X55 treatment (270, orange circle), and the number of metabolites deregulated in both CDA-depleted cells left untreated relative to control cells (125/228), and in X55-treated CDA-depleted cells relative to untreated CDA-depleted cells (125/270). In total 76 of these metabolites were deregulated in the same way, with 55 displaying an increase in levels and 21 displaying a decrease in levels, and 49 metabolites displaying deregulation in opposite directions

CDA is a major actor in pyrimidine metabolism, and several metabolomic pathways are known to be interconnected. We therefore investigated the metabolic pathways altered by CDA depletion and the reasons for which X55 preferentially targeted CDA-deficient tumor cells. We conducted a large-scale metabolomic study on HeLa control cells and CDA-depleted HeLa cells that were left untreated or treated with X55 (1 μM, 24 h, to generate a partial effect on the survival of CDA-depleted HeLa cells). Five biological replicates of each group were subjected to cell survival assays, cell cycle and western-blot analyses followed by metabolomic profiling. The survival and cell cycle progression of HeLa control cells were unaffected by X55 treatment, whereas X55 decreased the survival of CDA-depleted HeLa cells by 35%, and about 58% of these cells were blocked in S phase (more than twice the proportion in control cells) (Fig. 2a and b and Supplementary Fig. 3). Moreover, western-blot analyses revealed PARP-1 cleavage only in X55-treated CDA-depleted HeLa cells, indicating that X55 treatment induced apoptosis in these cells (Fig. 2c).

The abundance of 531 metabolites from eight large families, consisting mostly of lipids (36%), amino acids (28%) and nucleotides (10%) (Supplementary Table 1), was analyzed by Metabolon Inc. (USA). ANOVA contrasts were applied to identify metabolites for which levels differed significantly between the four experimental groups.

The pyrimidine metabolites cytidine and 2’-deoxycytidine were among the detectable metabolites differing in abundance between untreated CDA-deficient and CDA-proficient cells. These metabolites were present in very small amounts in CDA-expressing cells and were detected in significantly larger amounts in CDA-deficient cells (33-fold higher levels of cytidine and 1.7-fold higher levels of 2’-deoxycytidine in CDA-depleted HeLa cells than in control HeLa cells) (Fig. 2d). Uridine and 2’-deoxyuridine levels were significantly lower in CDA-depleted HeLa cells (3.4-fold lower and almost entirely absent, respectively) than in control cells (Fig. 2e). The abundance of these nucleotides was not affected by X55 treatment (Fig. 2d and e). Thus, CDA depletion led to an increase in cytidine and deoxycytidine levels and a decrease in uridine and 2’-deoxyuridine levels, as expected, validating our approach.

Global analyses of metabolite levels in the two isogenic cell lines, with and without X55 treatment, revealed that the levels of 228 metabolites differed significantly between CDA-depleted HeLa and control HeLa cells (*p* < 0.05) (Fig. 2f, Table 2 and Supplementary Table 2). Moreover, X55 treatment resulted in deregulation of the levels of 49 metabolites in control cells and 270 metabolites in CDA-depleted cells (Fig. 2f, Table 2 and Supplementary Table 3). Most of the metabolites deregulated by X55 treatment in control cells were different from those deregulated in CDA-depleted cells. Only 17 metabolites were deregulated in the same way in both types of cell by X55 treatment (names in bold in Supplementary Table 3). However, the levels of 125 metabolites were deregulated in both untreated CDA-depleted cells relative to control cells (125/228) and in X55-treated CDA-depleted cells relative to untreated CDA-depleted cells (125/270) (Fig. 2f and Supplementary Table 3). These metabolites included 76 that were deregulated in the same way in both sets of conditions, with deregulation thus aggravated by X55 treatment, 55 of which displayed an increase in levels with X55 treatment (in red in Supplementary Table 4), the other 21 displaying a decrease in levels (in green in Supplementary Table 4). Moreover, the levels of 49 compounds were deregulated the opposite directions in X55-treated CDA-depleted HeLa cells and untreated CDA-depleted HeLa cells (Fig. 2f, names in bold in Supplementary Table 4).

**Table 2:**
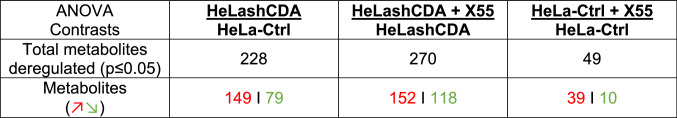
Summary of the numbers of metabolites with abundances differing significantly (*P* ≤ 0.05) between the two isogenic cell lines, with and without treatment with 1 μM X55 for 24 h

Two-way ANOVA identified metabolites with abundances differing significantly between the four experimental groups (Metabolon Inc.). The upregulated metabolites are shown in red, and the downregulated metabolites in green

Thus, 24 h after X55 treatment, CDA-depleted cells were blocked in the S-phase of the cell cycle and had initiated apoptosis, whereas this was not the case for control cells. Moreover, the metabolite profiles of CDA-depleted HeLa cells were clearly different from those of control HeLa cells. X55 treatment had a mild effect on control HeLa cells, whereas it significantly disturbed the metabolome of CDA-depleted HeLa cells, aggravating the deregulation of 76 metabolites. These findings suggest that X55 specificity is at least partly dependent on the concomitant depletion of CDA.

### The levels of 2-hydroxyglutarate, succinate and fumarate are significantly lower in CDA-depleted cells and are further decreased by X55 treatment

**Fig. 3:**
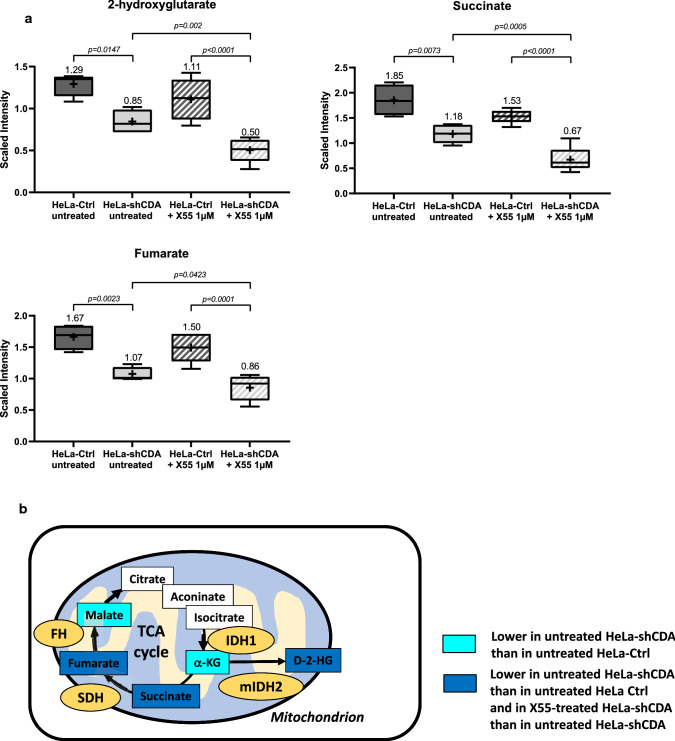
The levels of three oncometabolites are significantly reduced in CDA-depleted cells, and further reduced in these cells by X55 treatment. **a** Measurement of the levels of 2’-hydroxyglutarate, succinate and fumarate in HeLa-Ctrl cells (dark gray) and HeLa-shCDA cells (light gray), left untreated (block-shaded) or treated for 24 h with 1 µM X55 (hatched). The error bars represent means ± SD for four or five independent experiments. The significance of differences was assessed by two-way ANOVA. **b** Schematic representation of the TCA cycle, focusing on metabolites for which levels decreased only in CDA-depleted HeLa cells, but not in HeLa control cells (turquoise blue), and on the three mitochondrial oncometabolites (dark blue), succinate, fumarate and 2-hydroxyglutarate (2-HG), the levels of which were lower in CDA-depleted HeLa cells than in control HeLa cells, and in X55-treated CDA-depleted HeLa cells than in untreated CDA-depleted HeLa cells

CDA expression is downregulated in about 60% of cancer cells and tissues [13], mainly by promoter DNA methylation, suggesting that CDA loss may contribute to the growth of tumor cells. Many studies have reported that the accumulation of three mitochondrial oncometabolites, 2-hydroxyglutarate, succinate and fumarate, drives the initiation and progression of cancers and is involved in drug resistance [2–5]. Indeed, mutations of the genes encoding the isocitrate dehydrogenase enzymes (IDH1 or IDH2), succinate dehydrogenase and fumarate hydratase, leading to accumulation of the 2-hydroxyglutarate, succinate and fumarate oncometabolites, have been reported in several types of cancer, resulting in a state of pseudohypoxia due to the inhibition of prolyl-hydroxylase enzymes [6, 17–20]. We therefore focused our analyses on these three oncometabolites. Unexpectedly, we found that their levels did not increase and that, on the contrary, they were significantly lower in CDA-depleted HeLa cells, than in control HeLa cells (Fig. 3a). Succinate and fumarate are TCA (tricarboxylic acid) cycle metabolites, whereas 2-hydroxyglutarate is derived from the TCA cycle metabolite α-ketoglutarate [6]. We therefore assessed the abundance of the other TCA cycle metabolites and key players in this pathway [6]. We found significantly lower levels of α-ketoglutarate and malate, with no change in the abundance of citrate, isocitrate and aconinate (Fig. 3b and Supplementary Fig. 4a). The abundances of other key metabolites for TCA cycle activity, such as glucose, pyruvate, acetyl-CoA, NAD^+^, NADH and FAD, were not affected by CDA depletion (Supplementary Fig. 4b).

We then analyzed the consequences of X55 treatment for the abundance of these metabolites. X55 further decreased the levels of the three oncometabolites in CDA-depleted HeLa cells, but had no effect on their levels in control HeLa cells (Fig. 3a). Moreover, X55 treatment had no effect on any of the tested TCA cycle metabolites or key players of this pathway in control HeLa cells, whereas it significantly increased the levels of citrate, isocitrate, aconinate, pyruvate and acetyl-CoA and decreased the levels of α-ketoglutarate and NADH in CDA-depleted HeLa cells, with no effect on the abundance of malate, NAD^+^ and FAD (Supplementary Fig. 4a and b).

Thus, CDA depletion in HeLa cells led to a significant decrease in the levels of the three oncometabolites studied. Moreover, X55 treatment had no effect on the abundance of any TCA cycle metabolite or key player of this pathway in control HeLa cells, but it further decreased the amounts of the three oncometabolites considered in CDA-depleted cells and significantly deregulated the abundance of most of the TCA cycle metabolites in these cells. These major changes in the abundance of TCA cycle metabolites in CDA-deficient HeLa cells raise the question of a potential mitochondrial dysfunction in CDA-deficient tumors and reveal, for the first time, an unexpected link between pyrimidine pool metabolism, mitochondrial function and energy metabolism.

### X55 treatment strongly downregulates *MAPT* expression in X55-sensitive tumor cells

**Fig. 4:**
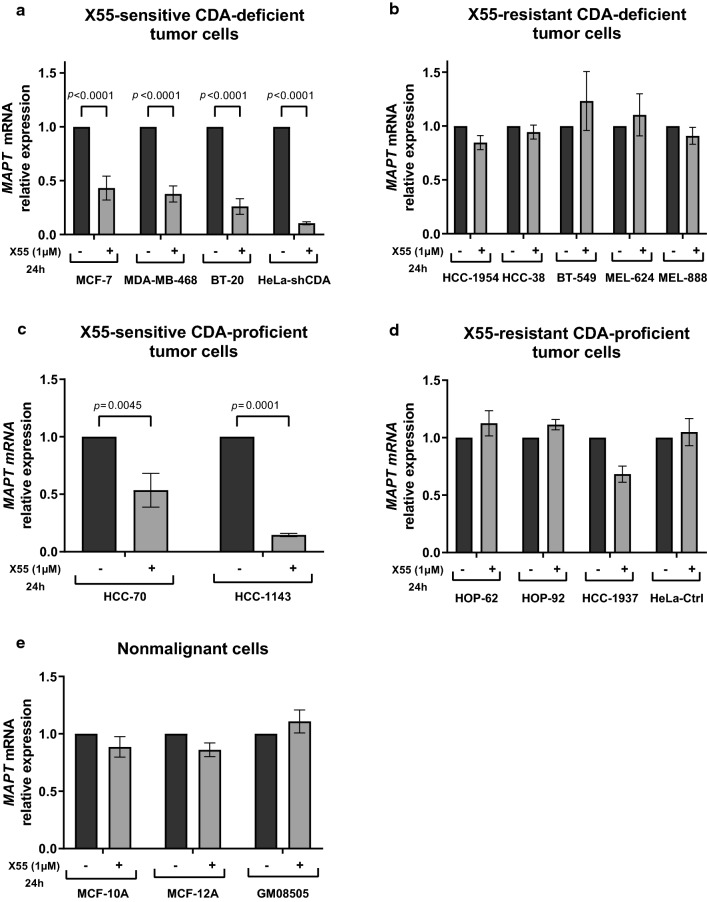
X55 treatment induces a strong downregulation of *MAPT* expression in X55-sensitive tumor cells. **a, b, c, d, e** *MAPT* expression was monitored by RT-qPCR in X55-sensitive CDA-deficient cells (**a**), X55-resistant CDA-deficient cells (**b**), X55-sensitive CDA-proficient cells (**c**), X55-resistant CDA-proficient cells (**d**), nonmalignant cells (**e**), left untreated (black bars) or treated with 1 µM X55 for 24 h (gray bars). *B2M* and *TBP* were used as housekeeping genes for normalization. The significance of differences was assessed in two-tailed paired Student’s *t*-tests. The error bars represent means ± SD for at least three independent experiments. *P* < 0.05 was considered statistically significant

We previously reported a synthetic lethal interaction between cytidine deaminase and Tau deficiencies and demonstrated that Tau is crucial for the survival of CDA-deficient cells, due to its role in ensuring the maintenance of genome integrity [12]. We therefore investigated the regulation of *MAPT* mRNA levels in response to X55 treatment in both CDA-deficient cells sensitive to X55 and in CDA-deficient cells resistant to X55. We treated CDA-deficient cells with X55 (1 μM, 24 h) and performed analyses of *MAPT* expression levels by reverse transcription-quantitative PCR (RT-qPCR) and cell survival assays in parallel (Fig. 4a and b, Supplementary Fig. 5a and b). We found a strong decrease in *MAPT* levels in response to X55, by more than 50%, in all X55-sensitive CDA-deficient tumor cells tested, whereas X55 treatment did not affect *MAPT* levels in X55-resistant CDA-deficient tumor cells (Fig. 4a and b and Supplementary Fig. 5a and b). We then performed the same experiments with CDA-proficient tumor cells and non-malignant cells. Again, a significant decrease in *MAPT* levels was observed in the two X55-sensitive CDA-proficient tumor cells, but not in X55-resistant CDA-proficient tumor cells (Fig. 4c and d and Supplementary Fig. 5c and d). X55 did not affect *MAPT* expression in nonmalignant cells, regardless of their CDA expression status (Fig. 4e and Supplementary Fig. 5e). Interestingly, the decrease in *MAPT* expression was not related to initial *MAPT* levels in the cells (Supplementary Fig. 5f).

Thus, *MAPT* levels decrease by more than 50% in all X55-sensitive tumor cell lines, regardless of their CDA expression status, but not in X55-resistant tumor or nonmalignant cell lines. These results reveal that a large decrease in *MAPT* levels can be used as an early predictive marker of cell sensitivity to X55.

## Discussion

We identified a naphthol derivative, X55, as a new potential anticancer molecule that preferentially targets CDA-deficient tumor cells. We also performed a large-scale metabolomic study with two isogenic tumor cell lines, CDA-depleted HeLa cells and control HeLa cells expressing endogenous CDA. We found that CDA-depletion per se resulted in significant changes in the levels of several metabolites relative to control cells. All metabolites for which abundance was modified by CDA depletion are presented in Supplementary Table 2. As expected, cytidine and deoxycytidine levels were higher, and uridine and deoxyuridine levels were lower in these cells. However, the levels of several other nucleotides of the pyrimidine pathway were also deregulated in CDA-depleted cells relative to control cells, with increases in cytidine triphosphate (CTP) and uridine triphosphate (UTP) levels, for example. The increase in CTP levels may be related to the high levels of cytidine available for phosphorylation, but the increase in UTP levels is counterintuitive, given the decrease in uridine levels, and is therefore difficult to interpret. Unexpectedly, CDA depletion also affected purine metabolism, the levels of most of amino acids and many other metabolic pathways (Supplementary Table 2). Global metabolite profiling in CDA-depleted HeLa cells yielded intriguing data that may open up new avenues for studies of the cellular regulation of nucleotide pools, in interaction with several metabolic pathways.

**Fig. 5:**
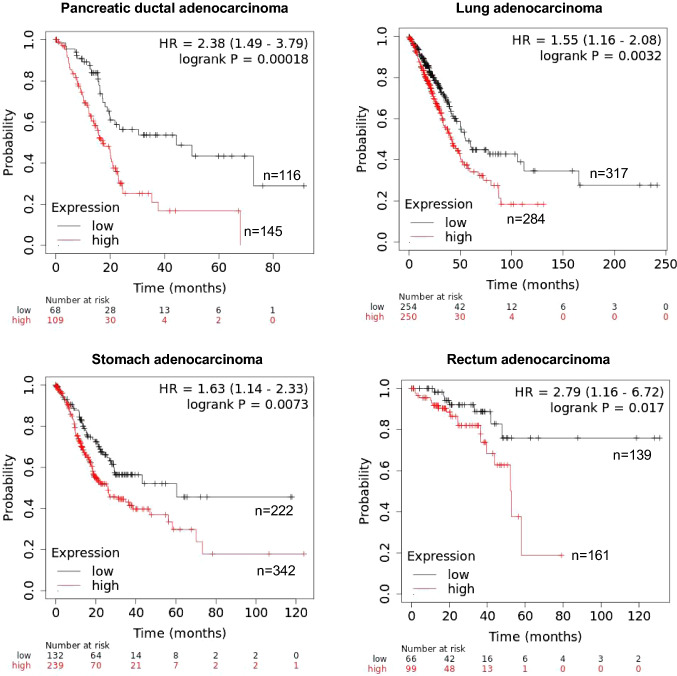
Kaplan–Meier curves showing the overall survival of patients with four different TCGA cancers. Patients were classified as having tumors with high or low levels of CDA expression, with the best available cutoff value used as an input parameter [31]. The data were obtained with pancancer data from the publicly available Kaplan–Meier Plotter database (https://kmplot.com/analysis/) [21]

TCA cycle metabolites are known to act as signaling molecules in several processes, including chromatin modification and DNA methylation, and the metabolomic reprogramming of cancer cells plays a major role in initiating tumorigenesis, cancer progression and resistance to anticancer therapies [2–6]. Three metabolites, 2-hydroxyglutarate, succinate and fumarate, have been identified as oncometabolites in many studies, because their accumulation in cancer cells has been shown to interfere with several pathways, including the regulation of epigenetic modifications [2–6]. We found that the levels of these three oncometabolites, and of other TCA cycle metabolites, were significantly lower in CDA-depleted cells than in control cells. Very little is known about the molecular mechanisms underlying decreases in oncometabolite levels and the cellular consequences of such decreases. Our results are the first to reveal such a decrease in oncometabolite levels. These results might suggest that CDA loss in tumors reflects an ultimate attempt by the cells to reverse the process of carcinogenesis. Moreover, in silico data analyses revealed that, for some cancers, such as pancreatic, lung, stomach or rectum adenocarcinomas, patients with tumors displaying little or no CDA expression have a better prognosis [21] (Fig. 5). DNA damage response mechanisms have been reported to act as a biological anticancer barrier when activated at early stages of cancer development [22–24]. We previously showed that CDA depletion in HeLa cells leads to the spontaneous activation of γ-H2AX and Chk2 [8]. In this context, the decrease in oncometabolite levels in CDA-depleted HeLa cells may reflect a mechanism for slowing carcinogenesis.

Our metabolomic analysis revealed that X55 treatment amplified some of the effects of CDA depletion, inducing a further decrease in the levels of the three oncometabolites, 2-hydroxyglutarate, succinate and fumarate. We also found that X55 induced significant changes in the levels of several TCA cycle metabolites in CDA-deficient HeLa cells, with no effect on the levels of these metabolites in control HeLa cells. These results suggest that the amplification of some of the cellular abnormalities associated with CDA deficiency by X55 treatment may contribute to the preferential targeting of CDA-deficient HeLa cells, and that the major changes in the abundance of several TCA cycle metabolites induced by X55 might contribute to the death of these cells. Our results therefore highlight, for the first time, a possible role for CDA in the mitochondrial metabolism. Identification of the molecular target of X55, which is underway in our laboratory, will undoubtedly facilitate a more profound interpretation of these metabolomic data.

Finally, based on our previous results showing the importance of Tau expression for the survival of CDA-deficient cells [12], we explored the regulation of *MAPT* mRNA levels in X55-sensitive and X55-resistant cells. Unexpectedly, we found that a common feature of all X55-sensitive cells was the induction, by X55, of a large decrease in *MAPT* levels, regardless of CDA expression status. These results raise major questions about (1) the mechanism underlying the death of some CDA-expressing tumor cells in association with the decrease in *MAPT* levels, and (2) the mechanism by which X55 induces a preferential decrease in *MAPT* expression levels in CDA-deficient tumor cells. We explored several hypotheses experimentally, but have obtained no clear answers as yet. Nevertheless, these results are of major importance in the context of a potential clinical application, because they reveal that decreases in *MAPT* levels are a reliable marker of cell sensitivity to X55.

In conclusion, we have identified a pharmacological tool, X55, the development of which could lead to a new anticancer treatment preferentially targeting CDA-deficient tumors. Our results also reveal an unexpected link between the pyrimidine pool, mitochondria and energy metabolism. CDA-depleted HeLa cells have many metabolic abnormalities and are characterized by a significant decrease in the abundance of several oncometabolites for which an accumulation has been implicated in both tumor initiation and progression, and in resistance to anticancer treatments. These findings suggest that CDA loss in tumors may reflect an ultimate attempt by the cells to slow down the process of tumorigenesis. Finally, we also identified a predictive marker of sensitivity to X55, which is essential in the context of a potential clinical application.

## Materials and methods

### Chemical library

The Institut Curie-CNRS chemical library (2004 version) contains 8560 compounds stored in 96-well microplates at a concentration of 10 mg/mL in DMSO (anhydrous dimethyl sulfoxide, Carl Roth, Karlsruhe, Germany). This chemical library was screened for compounds targeting CDA deficiency. Active compounds identified in the primary screen were retested with freshly made solutions.

### Automated screening

Automated screening of the chemical library was performed in white, 384-well microplates, with the automated platform of the CMBA (*Centre de Criblage pour des Molécules Bio-Actives*, Grenoble) screening facilities, as described by Prudent et al. [25]. CDA-proficient (HeLa-Ctrl) or CDA-deficient (HeLa-shCDA) cells were used to seed 384-well plates (25 µL per well, 8 × 10^4^ cells/mL), which were incubated for 24 h at 37 °C under an atmosphere containing 5% CO_2_ in a damp chamber. We then added 5 µL of each compound at the concentrations indicated (final concentration of 1–10 µM), in duplicate, with a final DMSO (compound vehicle) concentration of 0.2%. DMSO was used alone as a positive control, and the absence of cells for seeding was used as a negative control. Plates were incubated for an additional 24 h. Cell viability was then quantified with the CellTiter-Glo^®^ Luminescent Cell Viability Assay (Promega Corp.) with luminescence quantified using a Tecan Infinite M100 reader. Data were analyzed with in-house TAMIS software, as follows: each crude signal value was normalized according to percent viability, based on the mean values of the positive and negative controls of the corresponding microplate, considered to correspond to 0% and 100% cytotoxicity, respectively. For the primary screen, the compounds for which the difference in viability between CDA-deficient and CDA-proficient cells was the greatest were selected as primary hits. For the secondary screen, hit selection was restricted to compounds with confirmed viability-reducing activity in CDA-deficient cells.

### X55 (8-hydroxy-4-methyoxy-1-naphthalene-carboxaldehyde) synthesis

C_12_H_10_O_3_ MW = 202.209 g mol^−1^

The X55 compound was synthesized according to standard procedures. The starting material for 8-hydroxy-4-methyoxy-1-naphthalene-carboxaldehyde was a commercially available 1,5-dihydroxynaphthalene protected as the *O*-methylated derivative [26]. Using procedures described by the team of Rapoport [27], the formyl group was introduced under Vilsmeier–Haack conditions. Naphthaldehyde was selectively demethylated at position 8, by treatment with boron tribromide in dichloromethane at − 60 °C, to generate 8-hydroxy-4-methyoxy-1-naphthalene-carboxaldehyde (X55) with a yield of 70%, in three steps.

### Spectroscopic analysis of X55

Proton (^1^H) NMR spectroscopy was performed on a Bruker Avance 300 (300 MHz for 1H), with TMS as an internal standard. Deuterated CDCl_3_ was purchased from Eurisotop. Chemical shifts are given in parts per million (ppm) (δ relative to the residual solvent peak for ^1^H). The following abbreviations were used: singlet (s), doublet (d), triplet (t) and multiplet (m). Purity was determined by high-performance liquid chromatography (HPLC) with an Alliance Waters system [Alliance Waters 2695 (pump) and Waters 2998 (photodiode array detector)] with a Waters XBridge C-18 column, and a particle size of 3.5 µm (3.0 mm × 100 mm).^1^H NMR (300 MHz, CDCl_3_) 12.16 (s, 1H), 9.63 (s, 1H), 7.98 (d, J = 8.0 Hz, 1H), 7.86 (d, J = 8.0 Hz, 1H), 7.49 (t, J = 8.0 Hz, 1H), 7.19 (d,J = 8.0 Hz, 1H), 6.89 (d,J = 8.0 Hz, 1H), 4.12 (s, 3H) HPLC purity = 100%; LRMS (ESI–MS) m/z = 202.8 [M + H]^+^.

### Cell culture

We used 34 cancer cell lines in this study (Table 1): 17 breast cancer cell lines from the Translational Research Department of the Curie Institute (Paris, France; ZR75-1, T47D, HCC-1428, BT-474, MCF-7, MDA-MB-468, MDA-MB-231, MDA-MB-436, HCC-38, HCC-70, HCC-1187, HCC-1937, HCC-1143, BT-20, BT-549, HCC-1954, and Hs578T), five nonmalignant cell lines (MCF-10A, MCF-12A, MRC-5 (from Dr Pierre-Marie Girard), GM8505B, HEK-293T), four lung cancer cell lines (H522, H23, HOP-92, and HOP-62) and two ovarian cancer cell lines (IGROV-1 and SKOV-3) from the NCI (Rockville, MD) [28], four melanoma cell lines (A2058, A375, MEL888, MEL624) from Dr. Stephan Vagner's laboratory (UMR3348 CNRS, Curie Institute), and two cervical cancer cell lines (HeLa-Ctrl and HeLa-shCDA), transfected with an empty pGIPZ vector or with the same vector encoding a short hairpin RNA sequence directed against CDA, respectively, as previously described [8]. In brief, all the breast cell lines were authenticated at the Translational Research Department of the Curie Institute, by the standard DNA microsatellite short tandem repeat (STR) method. Authenticity of melanoma cell lines was assessed by comparing the short tandem repeat profile generated with the profiles present in the Deutsche Sammlung von Mikroorganismen und Zellkulturen. Lung and ovarian cancer cell lines were authenticated at the NCI with the standard DNA microsatellite STR method [28]. The isogenic HeLa-Ctrl/ HeLa-shCDA lines were established in our laboratory and are routinely checked for several phenotypic characteristics, including sister chromatid exchange and ultrafine anaphase bridge frequencies [8, 29].

All cells were routinely checked for the absence of mycoplasma and were maintained in the recommended media (see Supplementary Material S1).

### Cell viability assay

Depending on the cell line used and the duration of the experiment, cells were plated at a density between 2 × 10^3^ and 8 × 10^3^ well in 96-well microplates, in triplicate. After 24 h, cells were left untreated or were treated with 1 μM X55, or at concentrations ranging from 0.001 to 10 µM. After 24 or 72 h, cell viability was assessed with 3-(4,5-dimethyl-2-thiazolyl)-2,5 diphenyl-2H-tetrazolium bromide (MTT; Life Technologies). X55 cytotoxicity was evaluated by calculating the percentage cell viability normalized against the corresponding controls for each set of conditions. For the experiment presented in Supplementary Fig. 2, cells were subjected to pretreatment with 100 μM tetrahydrouridine (THU) (Calbiochem; 584222) for 96 h (2 × 48 h), and were then left untreated or were treated with 1 μM X55 for 24 h. Their viability was then assessed.

### Flow cytometry analysis

Cells were detached by treatment with Accutase (Sigma), immediately washed in 1 × PBS, fixed in cold 70% ethanol and stored at − 20 °C overnight. Cells were then washed twice with ice-cold 1 × PBS and incubated with Vindelov solution (Tris HCl, pH 7.6, 3.5 mM; NaCl 10 mM, propidium iodide 50 μg mL^−1^; 0.1% NP40; 20 μg mL^−1^ RNAse) for 30 min in the dark. Finally, cell cycle analysis was performed with a FACSCanto II machine from BD Biosciences. The percentage of cells in S phase was determined with the Dean-Jett-Fox model in FlowJo software.

### Immunoblotting

Immunoblotting was performed as previously described [11]. The following antibodies were used for detection: rabbit anti-PARP-1 (#ALX-210-302-R100, Enzo Life Sciences, 1:4000), rabbit anti-CDA (#ab56053, Abcam, dilution 1:500), rabbit anti-β-actin (#A2066, Sigma-Aldrich, 1:5000) and a horseradish peroxidase (HRP)-conjugated goat anti-rabbit IgG (#A9169, Sigma-Aldrich, 1:5000). Bands were visualized by chemiluminescence (Clarity Western ECL Substrate, Bio-Rad), with a ChemiDoc XRS + Molecular Imager and Image Lab Software (Bio-Rad).

### Immunofluorescence microscopy

Immunofluorescence staining and analysis were performed as previously described [30]. Primary and secondary antibodies were used at the following concentrations: rabbit anti-PICH antibody (H00054821-D01P, Abnova, dilution 1:150), and goat anti-rabbit Alexa Fluor 555 (#A21429, Life Technologies, dilution 1:500). Cell images were acquired with a 3-D deconvolution imaging system consisting of a Leica DM RXA microscope equipped with a piezoelectric translator (PIFOC; PI) placed at the base of a 63 × PlanApo N.A. 1.4 objective, and a CoolSNAP HQ interline CCD camera (Photometrics). Stacks of conventional fluorescence images were collected automatically at a Z-distance of 0.2 µm (Metamorph software; Molecular Devices).

### Reverse transcription and real-time quantitative PCR

Reverse transcription and real-time quantitative PCR were performed as previously described [12]. Total RNA was extracted with the NucleoSpin RNA kit (Macherey–Nagel). RNA quality was assessed with a NanoDrop 2000 spectrophotometer, and cDNAs were synthesized with the iScript Advanced cDNA synthesis kit (Bio-Rad) and 2 μg of RNA. Real-time PCR was performed with the cDNA template (1/20 dilution), iQ SYBR Green Supermix (Bio-Rad), and 300 nM forward and reverse primers. Amplification was performed with the CFX96 detection system (Bio-Rad). The relative quantities of the *MAPT* and *CDA* cDNAs were normalized against two reference genes (*B2M* and *TBP*) or *GAPDH*. The primer sequences are provided in Supplementary Material S2.

### Large-scale metabolomic analysis

The metabolomic analysis was performed by Metabolon, Inc (Durham, NC, www.metabolon.com). The isogenic HeLa-Ctrl and HeLa-shCDA cell lines were cultured and collected according to the sample preparation guidelines provided by Metabolon. Pellets of 12 × 10^6^ cells for each set of conditions were flash-frozen and sent to Metabolon for standard solvent extraction and global metabolomic analysis.

In total, 531 metabolites of known identity were analyzed. Following normalization against protein concentration in a Bradford assay, logarithmic transformation was performed and any missing values were imputed, from the minimum observed value for each compound, ANOVA contrasts were used to identify metabolites differing significantly in abundance between experimental groups, and two-way ANOVA was used to identify metabolites displaying significant interactions and main effects for the experimental parameters of treatment and genotype. One sample from the control HeLa group (without X55 treatment) was considered to be an outlier and was excluded from the statistical analysis.

### Statistical analysis

At least three independent experiments were performed to generate each dataset. The statistical significance of differences in mRNA levels was determined in two-tailed paired *t*-tests.

Metabolomic analyses were performed by Metabolon Inc. The false discovery rate (*q*-value) was estimated, to take into account the multiple comparisons that normally occur in metabolomics-based studies. The *q*-value describes the false discovery rate; a low *q*-value (*q* < 0.10) indicates a high degree of confidence in the results. Other statistical analyses were performed with GraphPad Prism 9.

## Supplementary Information

Below is the link to the electronic supplementary material.

Supplementary file1 (XLSX 176 KB)

Supplementary file2 (DOCX 52 KB)

Supplementary file3 (DOCX 73 KB)

Supplementary file4 (DOCX 43 KB)

Supplementary file5 (PDF 964 KB)

## Data Availability

Raw metabolomic data are presented in Supplementary Table 1.
