# Supplementary material for: Cytidine deaminase deficiency in tumor cells is associated with sensitivity to a naphthol derivative and a decrease in oncometabolite levels
Source: Cell Mol Life Sci. 2022 Aug 4;79(8):465. doi: 10.1007/s00018-022-04487-9 (PMC9352748; doi:10.1007/s00018-022-04487-9)
Supplement: Supplementary file 2 — Supplementary file2 (DOCX 52 KB) [file 18_2022_4487_MOESM2_ESM.docx]

Supplementary table 2

| Metabolite name | Super pathway | Sub pathway | HeLa-shCDA HeLa-Ctrl |
| --- | --- | --- | --- |
| N-acetylserine | Amino acid | Glycine, serine and threonine metabolism | **0.61** |
| N-acetylasparagine | Amino acid | Alanine and aspartate metabolism | **0.52** |
| N-acetylaspartate (NAA) | Amino acid | Alanine and aspartate metabolism | **0.76** |
| 5-hydroxylysine | Amino acid | Lysine metabolism | **0.34** |
| 2-aminoadipate | Amino acid | Lysine metabolism | **0.58** |
| Glutarate (pentanedioate) | Amino acid | Lysine metabolism | **0.6** |
| Tryptophan betaine | Amino acid | Tryptophan metabolism | **0.43** |
| Cystathionine | Amino acid | Methionine, cysteine, SAM and taurine metabolism | **0.67** |
| Cysteine | Amino acid | Methionine, cysteine, SAM and taurine metabolism | **0.48** |
| Cysteine sulfinic acid | Amino acid | Methionine, cysteine, SAM and taurine metabolism | **0.48** |
| Creatine | Amino acid | Creatine metabolism | **0.46** |
| Creatinine | Amino acid | Creatine metabolism | **0.73** |
| 1-methylguanidine | Amino acid | Guanidino and acetamido metabolism | **0.56** |
| Cysteine-glutathione disulfide | Amino acid | Glutathione metabolism | **0.38** |
| 5-oxoproline | Amino acid | Glutathione metabolism | **0.71** |
| Gamma-glutamylalanine | Peptide | Gamma-glutamyl amino acid | **0.22** |
| Gamma-glutamylglutamate | Peptide | Gamma-glutamyl amino acid | **0.47** |
| Gamma-glutamylglutamine | Peptide | Gamma-glutamyl amino acid | **0.2** |
| Gamma-glutamylhistidine | Peptide | Gamma-glutamyl amino acid | **0.6** |
| Gamma-glutamylmethionine | Peptide | Gamma-glutamyl amino acid | **0.47** |
| Fructose-6-phosphate | Carbohydrate | Glycolysis, gluconeogenesis, and pyruvate metabolism | **0.6** |
| Glycerate | Carbohydrate | Glycolysis, gluconeogenesis, and pyruvate metabolism | **0.12** |
| Ribose 1-phosphate | Carbohydrate | Pentose phosphate pathway | **0.68** |
| Ribose | Carbohydrate | Pentose metabolism | **0.04** |
| Ribitol | Carbohydrate | Pentose metabolism | **0.42** |
| Fructose | Carbohydrate | Fructose, mannose and galactose metabolism | **0.56** |
| Glucosamine-6-phosphate | Carbohydrate | Aminosugar metabolism | **0.36** |
| N-acetylglucosamine 6-phosphate | Carbohydrate | Aminosugar metabolism | **0.48** |
| Alpha-ketoglutarate | Energy | TCA cycle | **0.59** |
| Succinate | Energy | TCA cycle | **0.64** |
| Fumarate | Energy | TCA cycle | **0.65** |
| Malate | Energy | TCA cycle | **0.74** |
| 2-methylcitrate/homocitrate | Energy | TCA cycle | **0.54** |
| Phosphate | Energy | Oxidative phosphorylation | **0.68** |
| Myristoleate (14:1n5) | Lipid | Long-chain fatty acid | **0.76** |
| Palmitoleate (16:1n7) | Lipid | Long-chain fatty acid | **0.6** |
| 10-heptadecenoate (17:1n7) | Lipid | Long-chain fatty acid | **0.47** |
| Oleate/vaccenate (18:1) | Lipid | Long-chain fatty acid | **0.67** |
| Linoleate (18:2n6) | Lipid | Polyunsaturated fatty acid (n3 and n6) | **0.59** |
| Linolenate [alpha or gamma; (18:3n3 or 6)] | Lipid | Polyunsaturated fatty acid (n3 and n6) | **0.59** |
| 15-methylpalmitate | Lipid | Fatty acid, branched | **0.62** |
| 2-hydroxyglutarate | Lipid | Fatty acid, dicarboxylate | **0.65** |
| Linoleoyl ethanolamide | Lipid | Endocannabinoid | **0.48** |
| Choline | Lipid | Phospholipid metabolism | **0.52** |
| Glycerophosphorylcholine (GPC) | Lipid | Phospholipid metabolism | **0.42** |
| Trimethylamine N-oxide | Lipid | Phospholipid metabolism | **0.56** |
| 1-stearoyl-2-oleoyl-GPE (18:0/18:1) | Lipid | Phospholipid metabolism | **0.7** |
| Glycerol | Lipid | Glycerolipid metabolism | **0.71** |
| 1-oleoyl-3-linoleoyl-glycerol (18:1/18:2) | Lipid | Diacylglycerol | **0.76** |
| 1-palmitoleoyl-3-oleoyl-glycerol (16:1/18:1) | Lipid | Diacylglycerol | **0.77** |
| Sphingomyelin (d18:2/23:0, d18:1/23:1, d17:1/24:1) | Lipid | Sphingolipid metabolism | **0.8** |
| 7-dehydrocholesterol | Lipid | Sterol | **0.3** |
| Inosine | Nucleotide | Purine metabolism, (hypo)xanthine/inosine-containing | **0.27** |
| Hypoxanthine | Nucleotide | Purine metabolism, (hypo)xanthine/inosine-containing | **0.31** |
| Xanthine | Nucleotide | Purine metabolism, (hypo)xanthine/inosine-containing | **0.39** |
| Xanthosine | Nucleotide | Purine metabolism, (hypo)xanthine/inosine-containing | **0.19** |
| 2'-deoxyinosine | Nucleotide | Purine metabolism, (hypo)xanthine/inosine-containing | **0.28** |
| N1-methyladenosine | Nucleotide | Purine metabolism, adenine-containing | **0.65** |
| N6-carbamoylthreonyladenosine | Nucleotide | Purine metabolism, adenine-containing | **0.58** |
| N6-succinyladenosine | Nucleotide | Purine metabolism, adenine-containing | **0.08** |
| Guanosine | Nucleotide | Purine metabolism, guanine-containing | **0.25** |
| 2'-deoxyguanosine | Nucleotide | Purine metabolism, guanine-containing | **0.57** |
| Orotate | Nucleotide | Pyrimidine metabolism, orotate-containing | **0.02** |
| Uridine | Nucleotide | Pyrimidine metabolism, uracil-containing | **0.29** |
| Uracil | Nucleotide | Pyrimidine metabolism, uracil-containing | **0.31** |
| 5-methyluridine (ribothymidine) | Nucleotide | Pyrimidine metabolism, uracil-containing | **0.41** |
| 2'-deoxyuridine | Nucleotide | Pyrimidine metabolism, uracil-containing | **0.04** |
| Thymidine 5'-monophosphate | Nucleotide | Pyrimidine metabolism, thymine-containing | **0.66** |
| Thymidine | Nucleotide | Pyrimidine metabolism, thymine-containing | **0.09** |
| Thymine | Nucleotide | Pyrimidine metabolism, thymine-containing | **0.28** |
| Methylphosphate | Nucleotide | Purine and pyrimidine metabolism | **0.29** |
| 3'-dephosphocoenzyme A | Cofactors and vitamins | Pantothenate and CoA metabolism | **0.18** |
| 5-methyltetrahydrofolate (5MeTHF) | Cofactors and vitamins | Folate metabolism | **0.65** |
| Pterin | Cofactors and vitamins | Pterin metabolism | **0.76** |
| Pyridoxamine | Cofactors and vitamins | Vitamin B6 metabolism | **0.83** |
| Genistein | Xenobiotics | Food component/plant | **0.61** |
| Gluconate | Xenobiotics | Food component/plant | **0.52** |
| S-carboxymethyl-L-cysteine | Xenobiotics | Drug | **0.59** |
| 2-piperidinemethanol | Xenobiotics | Chemical | **0.24** |
| Threonine | Amino acid | Glycine, serine and threonine metabolism | **1.48** |
| Phosphothreonine | Amino acid | Glycine, serine and threonine metabolism | **2.74** |
| Alanine | Amino acid | Alanine and aspartate metabolism | **1.32** |
| Asparagine | Amino acid | Alanine and aspartate metabolism | **1.3** |
| Glutamate, gamma-methyl ester | Amino acid | Glutamate metabolism | **1.96** |
| Histidine | Amino acid | Histidine metabolism | **1.77** |
| N-acetylhistidine | Amino acid | Histidine metabolism | **1.48** |
| 1-methylhistidine | Amino acid | Histidine metabolism | **2.22** |
| 3-methylhistidine | Amino acid | Histidine metabolism | **1.66** |
| Imidazole lactate | Amino acid | Histidine metabolism | **1.91** |
| N6,N6,N6-trimethyllysine | Amino acid | Lysine metabolism | **1.47** |
| Pipecolate | Amino acid | Lysine metabolism | **1.85** |
| Cadaverine | Amino acid | Lysine metabolism | **1.79** |
| N-acetyl-cadaverine | Amino acid | Lysine metabolism | **1.6** |
| Phenylalanine | Amino acid | Phenylalanine and tyrosine metabolism | **1.5** |
| Phenyllactate (PLA) | Amino acid | Phenylalanine and tyrosine metabolism | **2.55** |
| Tyrosine | Amino acid | Phenylalanine and tyrosine metabolism | **1.59** |
| 4-hydroxyphenylpyruvate | Amino acid | Phenylalanine and tyrosine metabolism | **2.58** |
| 3-(4-hydroxyphenyl)lactate | Amino acid | Phenylalanine and tyrosine metabolism | **2.71** |
| Tryptophan | Amino acid | Tryptophan metabolism | **1.74** |
| Indolelactate | Amino acid | Tryptophan metabolism | **2.03** |
| 5-hydroxyindoleacetate | Amino acid | Tryptophan metabolism | **1.58** |
| C-glycosyltryptophan | Amino acid | Tryptophan metabolism | **1.76** |
| Leucine | Amino acid | Leucine, isoleucine and valine metabolism | **1.53** |
| Beta-hydroxyisovalerate | Amino acid | Leucine, isoleucine and valine metabolism | **1.39** |
| Beta-hydroxyisovaleroylcarnitine | Amino acid | Leucine, isoleucine and valine metabolism | **1.41** |
| 5-methylnorleucine | Amino acid | Leucine, isoleucine and valine metabolism | **1.92** |
| Isoleucine | Amino acid | Leucine, isoleucine and valine metabolism | **1.5** |
| 2-methylbutyrylcarnitine (C5) | Amino acid | Leucine, isoleucine and valine metabolism | **1.76** |
| Valine | Amino acid | Leucine, isoleucine and valine metabolism | **1.68** |
| 3-hydroxyisobutyrate | Amino acid | Leucine, isoleucine and valine metabolism | **1.46** |
| Alpha-hydroxyisocaproate | Amino acid | Leucine, isoleucine and valine metabolism | **5.18** |
| Methionine | Amino acid | Methionine, cysteine, SAM and taurine metabolism | **1.6** |
| Methionine sulfone | Amino acid | Methionine, cysteine, SAM and taurine metabolism | **2.24** |
| Methionine sulfoxide | Amino acid | Methionine, cysteine, SAM and taurine metabolism | **2.25** |
| N-acetylmethionine sulfoxide | Amino acid | Methionine, cysteine, SAM and taurine metabolism | **1.57** |
| S-adenosylmethionine (SAM) | Amino acid | Methionine, cysteine, SAM and taurine metabolism | **1.29** |
| S-adenosylhomocysteine (SAH) | Amino acid | Methionine, cysteine, SAM and taurine metabolism | **1.24** |
| 2-aminobutyrate | Amino acid | Methionine, cysteine, SAM and taurine metabolism | **2.09** |
| Ornithine | Amino acid | Urea cycle, arginine and proline metabolism | **1.46** |
| Citrulline | Amino acid | Urea cycle, arginine and proline metabolism | **3.3** |
| Homoarginine | Amino acid | Urea cycle, arginine and proline metabolism | **1.57** |
| Homocitrulline | Amino acid | Urea cycle, arginine and proline metabolism | **2.07** |
| N-delta-acetylornithine | Amino acid | Urea cycle, arginine and proline metabolism | **1.93** |
| Trans-4-hydroxyproline | Amino acid | Urea cycle, arginine and proline metabolism | **1.39** |
| Guanidinoacetate | Amino acid | Creatine metabolism | **2.32** |
| Putrescine | Amino acid | Polyamine metabolism | **2.07** |
| Spermine | Amino acid | Polyamine metabolism | **2.72** |
| Spermidine | Amino acid | Polyamine metabolism | **1.7** |
| 5-methylthioadenosine (MTA) | Amino acid | Polyamine metabolism | **1.44** |
| N-acetylputrescine | Amino acid | Polyamine metabolism | **2.1** |
| Glutathione, reduced (GSH) | Amino acid | Glutathione metabolism | **1.69** |
| Glutathione, oxidized (GSSG) | Amino acid | Glutathione metabolism | **1.69** |
| S-methylglutathione | Amino acid | Glutathione metabolism | **2.25** |
| Ophthalmate | Amino acid | Glutathione metabolism | **26** |
| Gamma-glutamylglycine | Peptide | Gamma-glutamyl amino acid | **1.76** |
| Gamma-glutamylisoleucine | Peptide | Gamma-glutamyl amino acid | **7.29** |
| Gamma-glutamylleucine | Peptide | Gamma-glutamyl amino acid | **6.84** |
| Gamma-glutamylphenylalanine | Peptide | Gamma-glutamyl amino acid | **2.06** |
| Gamma-glutamylthreonine | Peptide | Gamma-glutamyl amino acid | **14.75** |
| Gamma-glutamyltyrosine | Peptide | Gamma-glutamyl amino acid | **1.53** |
| Gamma-glutamylvaline | Peptide | Gamma-glutamyl amino acid | **15.34** |
| Alanylleucine | Peptide | Dipeptide | **1.59** |
| Glycylleucine | Peptide | Dipeptide | **1.31** |
| UDP-glucose | Carbohydrate | Nucleotide sugar | **1.3** |
| UDP-galactose | Carbohydrate | Nucleotide sugar | **1.35** |
| Caprylate (8:0) | Lipid | Medium-chain fatty acid | **3.04** |
| Nervonate (24:1n9) | Lipid | Long-chain fatty acid | **1.88** |
| Docosadienoate (22:2n6) | Lipid | Polyunsaturated fatty acid (n3 and n6) | **1.73** |
| Dihomo-linoleate (20:2n6) | Lipid | Polyunsaturated fatty acid (n3 and n6) | **1.72** |
| Butyrylcarnitine | Lipid | Fatty acid metabolism (also BCAA metabolism) | **1.63** |
| Acetylcarnitine | Lipid | Fatty acid metabolism (acyl carnitine) | **1.49** |
| Hexanoylcarnitine | Lipid | Fatty acid metabolism (acyl carnitine) | **1.5** |
| Octanoylcarnitine | Lipid | Fatty acid metabolism (acyl carnitine) | **2.25** |
| Decanoylcarnitine | Lipid | Fatty acid metabolism (acyl carnitine) | **1.69** |
| Laurylcarnitine | Lipid | Fatty acid metabolism (acyl carnitine) | **2.84** |
| Myristoylcarnitine | Lipid | Fatty acid metabolism (acyl carnitine) | **1.87** |
| Palmitoylcarnitine | Lipid | Fatty acid metabolism (acyl carnitine) | **1.44** |
| Stearoylcarnitine | Lipid | Fatty acid metabolism (acyl carnitine) | **1.59** |
| Myristoleoylcarnitine | Lipid | Fatty acid metabolism (acyl carnitine) | **1.51** |
| Deoxycarnitine | Lipid | Carnitine metabolism | **1.48** |
| Carnitine | Lipid | Carnitine metabolism | **2.12** |
| N-palmitoyltaurine | Lipid | Endocannabinoid | **1.94** |
| Inositol 1-phosphate (I1P) | Lipid | Inositol metabolism | **6.07** |
| Choline phosphate | Lipid | Phospholipid metabolism | **1.55** |
| 1-stearoyl-2-arachidonoyl-GPE (18:0/20:4) | Lipid | Phospholipid metabolism | **1.6** |
| 1,2-dioleoyl-GPG (18:1/18:1) | Lipid | Phospholipid metabolism | **1.37** |
| 1-palmitoyl-2-stearoyl-GPC (16:0/18:0) | Lipid | Phospholipid metabolism | **1.54** |
| 1,2-dioleoyl-GPS (18:1/18:1) | Lipid | Phospholipid metabolism | **1.46** |
| 1-palmitoyl-2-oleoyl-GPS (16:0/18:1) | Lipid | Phospholipid metabolism | **1.44** |
| 1-stearoyl-2-arachidonoyl-GPS (18:0/20:4) | Lipid | Phosphatidylserine (PS) | **1.91** |
| 1-arachidonoyl-GPE (20:4n6) | Lipid | Lysolipid | **2.08** |
| 1-stearoyl-GPS (18:0) | Lipid | Lysolipid | **1.33** |
| 1-palmitoyl-GPS (16:0) | Lipid | Lysolipid | **2.27** |
| 1-(1-enyl-stearoyl)-2-linoleoyl-GPE (P-18:0/18:2) | Lipid | Plasmalogen | **1.58** |
| 1-(1-enyl-stearoyl)-2-arachidonoyl-GPE (P-18:0/20:4) | Lipid | Plasmalogen | **1.45** |
| 1-(1-enyl-stearoyl)-GPE (P-18:0) | Lipid | Lysoplasmalogen | **1.34** |
| N-palmitoyl-sphinganine (d18:0/16:0) | Lipid | Sphingolipid metabolism | **1.49** |
| Sphingomyelin (d18:1/18:1, d18:2/18:0) | Lipid | Sphingolipid metabolism | **1.21** |
| Sphingomyelin (d18:1/14:0, d16:1/16:0) | Lipid | Sphingolipid metabolism | **1.26** |
| Sphingomyelin (d18:2/16:0, d18:1/16:1) | Lipid | Sphingolipid metabolism | **1.62** |
| Sphingomyelin (d18:1/15:0, d16:1/17:0) | Lipid | Sphingolipid metabolism | **1.35** |
| Glycosyl-N-stearoyl-sphingosine | Lipid | Sphingolipid metabolism | **1.23** |
| Glycosyl-N-palmitoyl-sphingosine | Lipid | Sphingolipid metabolism | **1.49** |
| Lactosyl-N-palmitoyl-sphingosine | Lipid | Sphingolipid metabolism | **3.43** |
| 3-hydroxy-3-methylglutarate | Lipid | Mevalonate metabolism | **1.38** |
| Cholate | Lipid | Primary bile acid metabolism | **1.51** |
| Glycochenodeoxycholate | Lipid | Primary bile acid metabolism | **1.55** |
| Inosine 5'-monophosphate (IMP) | Nucleotide | Purine metabolism, (hypo)xanthine/inosine containing | **18.07** |
| Adenosine 5'-diphosphate (ADP) | Nucleotide | Purine metabolism, adenine-containing | **6.08** |
| Adenosine 5'-monophosphate (AMP) | Nucleotide | Purine metabolism, adenine-containing | **3.35** |
| Adenosine 3'-monophosphate (3'-AMP) | Nucleotide | Purine metabolism, adenine-containing | **4.48** |
| Adenosine 3',5'-cyclic monophosphate (cAMP) | Nucleotide | Purine metabolism, adenine-containing | **2.09** |
| Adenylosuccinate | Nucleotide | Purine metabolism, adenine-containing | **4.98** |
| 2'-deoxyadenosine 5'-monophosphate | Nucleotide | Purine metabolism, adenine-containing | **3.36** |
| Guanosine 5'-triphosphate | Nucleotide | Purine metabolism, guanine-containing | **9.27** |
| Guanosine 5'- diphosphate (GDP) | Nucleotide | Purine metabolism, guanine-containing | **10.26** |
| Guanosine 5'- monophosphate (5'-GMP) | Nucleotide | Purine metabolism, guanine-containing | **5.33** |
| Uridine 5'-triphosphate (UTP) | Nucleotide | Pyrimidine metabolism, uracil-containing | **2.93** |
| Uridine 5'-diphosphate (UDP) | Nucleotide | Pyrimidine metabolism, uracil-containing | **2.82** |
| Uridine 5'-monophosphate (UMP) | Nucleotide | Pyrimidine metabolism, uracil-containing | **2.1** |
| Cytidine triphosphate | Nucleotide | Pyrimidine metabolism, cytidine-containing | **3.13** |
| Cytidine diphosphate | Nucleotide | Pyrimidine metabolism, cytidine-containing | **3.45** |
| Cytidine 5'-monophosphate (5'-CMP) | Nucleotide | Pyrimidine metabolism, cytidine-containing | **1.54** |
| Cytidine | Nucleotide | Pyrimidine metabolism, cytidine-containing | **33.41** |
| 5-methylcytidine | Nucleotide | Pyrimidine metabolism, cytidine-containing | **2.94** |
| 2'-deoxycytidine 5'-monophosphate | Nucleotide | Pyrimidine metabolism, cytidine-containing | **6.35** |
| 2'-deoxycytidine | Nucleotide | Pyrimidine metabolism, cytidine-containing | **1.71** |
| 1-methylnicotinamide | Cofactors and vitamins | Nicotinate and nicotinamide metabolism | **2.21** |
| Adenosine 5'-diphosphoribose (ADP-ribose) | Cofactors and vitamins | Nicotinate and nicotinamide metabolism | **1.43** |
| Riboflavin (Vitamin B2) | Cofactors and vitamins | Riboflavin metabolism | **1.35** |
| Phosphopantetheine | Cofactors and vitamins | Pantothenate and CoA metabolism | **2.14** |
| Thiamine monophosphate | Cofactors and vitamins | Thiamine metabolism | **5.27** |
| Pyridoxine (Vitamin B6) | Cofactors and vitamins | Vitamin B6 metabolism | **7.67** |
| Pyridoxal phosphate | Cofactors and vitamins | Vitamin B6 metabolism | **3.85** |
| Pyridoxate | Cofactors and vitamins | Vitamin B6 metabolism | **1.61** |
| Hippurate | Xenobiotics | Benzoate metabolism | **1.39** |
| 3-hydroxyhippurate | Xenobiotics | Benzoate metabolism | **1.41** |
| 4-vinylphenol sulfate | Xenobiotics | Benzoate metabolism | **1.51** |
| Ergothioneine | Xenobiotics | Food component/plant | **1.36** |
| Stachydrine | Xenobiotics | Food component/plant | **1.35** |
| Thymol sulfate | Xenobiotics | Food component/plant | **3.13** |
| Pyrraline | Xenobiotics | Food component/plant | **2.39** |
| Daidzein sulfate (2) | Xenobiotics | Food component/plant | **4.06** |
| Penicillin G | Xenobiotics | Drug | **1.92** |
| O-sulfo-L-tyrosine | Xenobiotics | Chemical | **1.48** |
| 2-aminophenol sulfate | Xenobiotics | Chemical | **1.49** |
| Phenol red | Xenobiotics | Chemical | **1.38** |
| Trizma acetate | Xenobiotics | Chemical | **1.31** |
