# Supplementary material for: Cytidine deaminase deficiency in tumor cells is associated with sensitivity to a naphthol derivative and a decrease in oncometabolite levels
Source: Cell Mol Life Sci. 2022 Aug 4;79(8):465. doi: 10.1007/s00018-022-04487-9 (PMC9352748; doi:10.1007/s00018-022-04487-9)
Supplement: Supplementary file 3 — Supplementary file3 (DOCX 73 KB) [file 18_2022_4487_MOESM3_ESM.docx]

Supplementary table 3

| Metabolite name | Super pathway | Sub pathway | HeLa-shCDA + X55 / HeLa-shCDA | HeLa-Ctrl + X55 /  HeLa-Ctrl |
| --- | --- | --- | --- | --- |
| **Fructose 1,6-bisphosphate** | Carbohydrate | Glycolysis, gluconeogenesis, and pyruvate metabolism | **2.4** | **2.29** |
| **Choline phosphate** | Lipid | Phospholipid metabolism | **1.39** | **1.34** |
| **1-stearoyl-2-oleoyl-GPE (18:0/18:1)** | Lipid | Phospholipid metabolism | **1.41** | **1.22** |
| **1-stearoyl-2-arachidonoyl-GPS (18:0/20:4)** | Lipid | Phosphatidylserine (PS) | **1.68** | **1.27** |
| **1-stearoyl-GPS (18:0)** | Lipid | Lysolipid | **1.29** | **1.25** |
| **1-(1-enyl-palmitoyl)-2-arachidonoyl-GPC (P-16:0/20:4)** | Lipid | Plasmalogen | **3.87** | **1.29** |
| **1-(1-enyl-stearoyl)-2-arachidonoyl-GPE (P-18:0/20:4)** | Lipid | Plasmalogen | **1.8** | **1.32** |
| **Sphingomyelin (d18:1/20:0, d16:1/22:0)** | Lipid | Sphingolipid metabolism | **1.37** | **1.26** |
| **Glycosyl-N-stearoyl-sphingosine** | Lipid | Sphingolipid metabolism | **2.32** | **1.23** |
| **Adenosine 5'-diphosphate (ADP)** | Nucleotide | Purine metabolism, adenine-containing | **1.54** | **2.19** |
| **Adenine** | Nucleotide | Purine metabolism, adenine-containing | **2.32** | **2.24** |
| **2'-deoxyadenosine 5'-monophosphate** | Nucleotide | Purine metabolism, adenine-containing | **2.49** | **2.84** |
| **Cytidine diphosphate** | Nucleotide | Pyrimidine metabolism, cytidine-containing | **2.85** | **1.5** |
| **Thymidine 5'-monophosphate** | Nucleotide | Pyrimidine metabolism, thymine-containing | **1.55** | **1.55** |
| **2-methylcitrate/homocitrate** | Energy | TCA cycle | **0.66** | **0.73** |
| **Sphingomyelin (d18:2/24:1, d18:1/24:2)** | Lipid | Sphingolipid metabolism | **0.69** | **0.71** |
| **N2,N2-dimethylguanosine** | Nucleotide | Purine metabolism, guanine-containing | **0.59** | **0.65** |
| Phosphothreonine | Amino acid | Glycine, serine and threonine metabolism | 1.43 | **1.69** |
| Cysteinylglycine | Amino acid | Glutathione metabolism | 0.92 | **1.22** |
| Phenylalanylalanine | Peptide | Dipeptide | 1.05 | **1.34** |
| Dihydroxyacetone phosphate (DHAP) | Carbohydrate | Glycolysis, gluconeogenesis, and pyruvate metabolism | 0.9 | **2.31** |
| Ribose 1-phosphate | Carbohydrate | Pentose Phosphate pathway | 0.92 | **1.57** |
| Laurylcarnitine | Lipid | Fatty acid metabolism (acyl carnitine) | 0.83 | **1.59** |
| Myristoylcarnitine | Lipid | Fatty acid metabolism (acyl carnitine) | **0.69** | **1.39** |
| Myristoleoylcarnitine | Lipid | Fatty acid metabolism (acyl carnitine) | **0.63** | **1.45** |
| 1-arachidonoyl-GPE (20:4n6) | Lipid | Lysolipid | 1.24 | **1.91** |
| 1-stearoyl-GPI (18:0) | Lipid | Lysolipid | 1.22 | **1.33** |
| 3-hydroxy-3-methylglutarate | Lipid | Mevalonate metabolism | 1.07 | **1.41** |
| Inosine 5'-monophosphate (IMP) | Nucleotide | Purine metabolism, (hypo)xanthine/inosine-containing | 1.36 | **2.41** |
| Adenosine 5'-monophosphate (AMP) | Nucleotide | Purine metabolism, adenine-containing | 0.94 | **1.92** |
| Adenylosuccinate | Nucleotide | Purine metabolism, adenine-containing | 0.77 | **4.92** |
| Guanosine 5'- diphosphate (GDP) | Nucleotide | Purine metabolism, guanine-containing | 1.75 | **3.61** |
| Guanosine 5'- monophosphate (5'-GMP) | Nucleotide | Purine metabolism, guanine-containing | 0.71 | **2.34** |
| Uridine 5'-monophosphate (UMP) | Nucleotide | Pyrimidine metabolism, uracil-containing | 1.01 | **1.83** |
| Cytidine 5'-monophosphate (5'-CMP) | Nucleotide | Pyrimidine metabolism, cytidine-containing | 1.18 | **1.45** |
| Cytidine | Nucleotide | Pyrimidine metabolism, cytidine-containing | 0.88 | **1.42** |
| 2'-deoxycytidine 5'-monophosphate | Nucleotide | Pyrimidine metabolism, cytidine-containing | 1.16 | **1.93** |
| Adenosine 5'-diphosphoribose (ADP-ribose) | Cofactors and vitamins | Nicotinate and nicotinamide metabolism | **0.72** | **1.53** |
| Riboflavin (Vitamin B2) | Cofactors and vitamins | Riboflavin metabolism | 1.11 | **1.25** |
| Thiamine monophosphate | Cofactors and vitamins | Thiamine metabolism | 1.15 | **2.56** |
| Pyridoxal phosphate | Cofactors and vitamins | Vitamin B6 metabolism | 1.02 | **1.42** |
| Ergothioneine | Xenobiotics | Food component/plant | **0.81** | **1.37** |
| Homocysteine | Amino acid | Methionine, cysteine, SAM and taurine metabolism | 0.98 | **0.45** |
| Glucose 6-phosphate | Carbohydrate | Glycolysis, gluconeogenesis, and pyruvate metabolism | 1.35 | **0.3** |
| Ribitol | Carbohydrate | Pentose metabolism | 0.87 | **0.71** |
| Glucosamine-6-phosphate | Carbohydrate | Aminosugar metabolism | 1.36 | **0.24** |
| N1-methyladenosine | Nucleotide | Purine metabolism, adenine-containing | 0.91 | **0.71** |
| Thymidine | Nucleotide | Pyrimidine metabolism, thymine-containing | 2.54 | **0.34** |
| Thymine | Nucleotide | Pyrimidine metabolism, thymine-containing | 0.89 | **0.69** |
| Betaine aldehyde | Amino acid | Glycine, serine and threonine metabolism | **2.42** | 1.35 |
| Serine | Amino acid | Glycine, serine and threonine metabolism | **1.6** | 1.01 |
| Asparagine | Amino acid | Alanine and aspartate metabolism | **1.53** | 1.05 |
| N-acetylaspartate (NAA) | Amino acid | Alanine and aspartate metabolism | **1.31** | 1.03 |
| Glutamine | Amino acid | Glutamate metabolism | **1.48** | 1.03 |
| N-acetylglutamate | Amino acid | Glutamate metabolism | **1.34** | 1.14 |
| Histidine | Amino acid | Histidine metabolism | **1.52** | 1.06 |
| 1-methylhistidine | Amino acid | Histidine metabolism | **1.52** | 1.1 |
| 3-methylhistidine | Amino acid | Histidine metabolism | **1.75** | 0.97 |
| Lysine | Amino acid | Lysine metabolism | **1.63** | 1.01 |
| N6-acetyllysine | Amino acid | Lysine metabolism | **1.73** | 1.02 |
| N6,N6,N6-trimethyllysine | Amino acid | Lysine metabolism | **1.71** | 1.15 |
| Phenylalanine | Amino acid | Phenylalanine and tyrosine metabolism | **1.48** | 1.03 |
| Tyrosine | Amino acid | Phenylalanine and tyrosine metabolism | **1.52** | 1.03 |
| O-methyltyrosine | Amino acid | Phenylalanine and tyrosine metabolism | **1.41** | 1.04 |
| Tryptophan | Amino acid | Tryptophan metabolism | **1.52** | 1.03 |
| 5-hydroxyindoleacetate | Amino acid | Tryptophan metabolism | **2.37** | 1.02 |
| Serotonin | Amino acid | Tryptophan metabolism | **2.09** | 0.96 |
| Leucine | Amino acid | Leucine, isoleucine and Valine metabolism | **1.48** | 1.04 |
| 5-methylnorleucine | Amino acid | Leucine, isoleucine and Valine metabolism | **1.53** | 1.05 |
| Isoleucine | Amino acid | Leucine, isoleucine and Valine metabolism | **1.47** | 1.03 |
| Valine | Amino acid | Leucine, isoleucine and Valine metabolism | **1.5** | 1.04 |
| Methionine | Amino acid | Methionine, cysteine, SAM and taurine metabolism | **1.48** | 1.01 |
| Methionine sulfone | Amino acid | Methionine, cysteine, SAM and taurine metabolism | **1.6** | 1.08 |
| Methionine sulfoxide | Amino acid | Methionine, cysteine, SAM and taurine metabolism | **1.55** | 1.07 |
| S-adenosylmethionine (SAM) | Amino acid | Methionine, cysteine, SAM and taurine metabolism | **1.35** | 1.07 |
| Cystathionine | Amino acid | Methionine, cysteine, SAM and taurine metabolism | **3.97** | 1.16 |
| Cysteine | Amino acid | Methionine, cysteine, SAM and taurine metabolism | **1.47** | 1.14 |
| N-acetylcysteine | Amino acid | Methionine, cysteine, SAM and taurine metabolism | **1.57** | 0.9 |
| S-methylcysteine | Amino acid | Methionine, cysteine, SAM and taurine metabolism | **4.35** | 1.01 |
| Arginine | Amino acid | Urea cycle, arginine and proline metabolism | **1.6** | 1.02 |
| Ornithine | Amino acid | Urea cycle, arginine and proline metabolism | **1.91** | 1.23 |
| Citrulline | Amino acid | Urea cycle, arginine and proline metabolism | **1.67** | 1.19 |
| Homoarginine | Amino acid | Urea cycle, arginine and proline metabolism | **1.74** | 0.99 |
| Homocitrulline | Amino acid | Urea cycle, arginine and proline metabolism | **1.74** | 1.06 |
| Dimethylarginine (SDMA + ADMA) | Amino acid | Urea cycle, arginine and proline metabolism | **1.32** | 0.94 |
| N-delta-acetylornithine | Amino acid | Urea cycle, arginine and proline metabolism | **1.59** | 1.04 |
| Pro-hydroxy-pro | Amino acid | Urea cycle, arginine and proline metabolism | **1.45** | 0.95 |
| Guanidinoacetate | Amino acid | Creatine metabolism | **3.91** | 0.96 |
| Spermine | Amino acid | Polyamine metabolism | **1.86** | 0.89 |
| N1,N12-diacetylspermine | Amino acid | Polyamine metabolism | **12.19** | 1.77 |
| Spermidine | Amino acid | Polyamine metabolism | **1.82** | 1.23 |
| 5-methylthioadenosine (MTA) | Amino acid | Polyamine metabolism | **1.36** | 1.13 |
| Cysteine-glutathione disulfide | Amino acid | Glutathione metabolism | **1.83** | 1.18 |
| 5-oxoproline | Amino acid | Glutathione metabolism | **1.45** | 1.01 |
| Gamma-glutamyl-epsilon-lysine | Peptide | Gamma-glutamyl amino acid | **1.58** | 1.12 |
| Glycylleucine | Peptide | Dipeptide | **1.27** | 1.21 |
| Glycylvaline | Peptide | Dipeptide | **1.44** | 1.19 |
| Prolylglycine | Peptide | Dipeptide | **1.41** | 1.26 |
| Valylglutamine | Peptide | Dipeptide | **1.67** | 1.2 |
| Fructose-6-phosphate | Carbohydrate | Glycolysis, gluconeogenesis, and pyruvate metabolism | **3.34** | 1 |
| Pyruvate | Carbohydrate | Glycolysis, gluconeogenesis, and pyruvate metabolism | **1.47** | 0.99 |
| Sedoheptulose-7-phosphate | Carbohydrate | Pentose phosphate pathway | **1.63** | 0.86 |
| UDP-glucose | Carbohydrate | Nucleotide sugar | **1.52** | 0.96 |
| UDP-galactose | Carbohydrate | Nucleotide sugar | **1.53** | 0.96 |
| UDP-glucuronate | Carbohydrate | Nucleotide sugar | **1.28** | 0.96 |
| Guanosine 5'-diphospho-fucose | Carbohydrate | Nucleotide sugar | **1.36** | 0.92 |
| UDP-N-acetylglucosamine | Carbohydrate | Nucleotide sugar | **1.78** | 1.06 |
| Cytidine 5'-monophospho-N-acetylneuraminic acid | Carbohydrate | Nucleotide sugar | **1.25** | 0.93 |
| N-acetyl-glucosamine 1-phosphate | Carbohydrate | Aminosugar metabolism | **1.83** | 1.11 |
| Citrate | Energy | TCA cycle | **1.6** | 1.08 |
| Isocitrate | Energy | TCA cycle | **2.17** | 0.85 |
| Eicosapentaenoate (EPA; 20:5n3) | Lipid | Polyunsaturated fatty acid (n3 and n6) | **1.84** | 0.99 |
| Docosapentaenoate (n3 DPA; 22:5n3) | Lipid | Polyunsaturated fatty acid (n3 and n6) | **1.99** | 1.08 |
| Docosahexaenoate (DHA; 22:6n3) | Lipid | Polyunsaturated fatty acid (n3 and n6) | **1.78** | 1.14 |
| Arachidonate (20:4n6) | Lipid | Polyunsaturated fatty acid (n3 and n6) | **2.06** | 0.98 |
| Acetyl CoA | Lipid | Fatty acid metabolism | **2.19** | 0.88 |
| Oleoyl ethanolamide | Lipid | Endocannabinoid | **1.22** | 0.99 |
| Stearoyl ethanolamide | Lipid | Endocannabinoid | **1.54** | 0.96 |
| N-oleoyltaurine | Lipid | Endocannabinoid | **2.46** | 1.32 |
| N-stearoyltaurine | Lipid | Endocannabinoid | **2.24** | 1.49 |
| N-palmitoyltaurine | Lipid | Endocannabinoid | **1.89** | 1.27 |
| Inositol 1-phosphate (I1P) | Lipid | Inositol metabolism | **1.73** | 1.08 |
| Choline | Lipid | Phospholipid metabolism | **1.32** | 0.97 |
| Cytidine 5'-diphosphocholine | Lipid | Phospholipid metabolism | **1.31** | 1.04 |
| Glycerophosphorylcholine (GPC) | Lipid | Phospholipid metabolism | **2.07** | 0.93 |
| Phosphoethanolamine | Lipid | Phospholipid metabolism | **2.19** | 1.01 |
| Glycerophosphoethanolamine | Lipid | Phospholipid metabolism | **1.75** | 1.17 |
| Glycerophosphoinositol | Lipid | Phospholipid metabolism | **1.58** | 0.99 |
| 1-palmitoyl-2-oleoyl-GPC (16:0/18:1) | Lipid | Phospholipid metabolism | **1.21** | 1.03 |
| 1-stearoyl-2-arachidonoyl-GPC (18:0/20:4) | Lipid | Phospholipid metabolism | **2.45** | 1.22 |
| 1-palmitoyl-2-linoleoyl-GPC (16:0/18:2) | Lipid | Phospholipid metabolism | **1.81** | 1.14 |
| 1-stearoyl-2-oleoyl-GPC (18:0/18:1) | Lipid | Phospholipid metabolism | **1.41** | 1.14 |
| 1-stearoyl-2-oleoyl-GPI (18:0/18:1) | Lipid | Phospholipid metabolism | **1.63** | 1 |
| 1-palmitoyl-2-arachidonoyl-GPC (16:0/20:4) | Lipid | Phospholipid metabolism | **1.46** | 1.12 |
| 1-stearoyl-2-arachidonoyl-GPI (18:0/20:4) | Lipid | Phospholipid metabolism | **1.75** | 1.17 |
| 1-oleoyl-2-linoleoyl-GPC (18:1/18:2) | Lipid | Phospholipid metabolism | **1.78** | 1.1 |
| 1-palmitoyl-2-stearoyl-GPC (16:0/18:0) | Lipid | Phospholipid metabolism | **1.39** | 1.21 |
| 1,2-dioleoyl-GPE (18:1/18:1) | Lipid | Phospholipid metabolism | **1.33** | 1.11 |
| 1,2-dioleoyl-GPS (18:1/18:1) | Lipid | Phospholipid metabolism | **1.36** | 1.02 |
| 1-oleoyl-2-linoleoyl-GPE (18:1/18:2) | Lipid | Phospholipid metabolism | **1.44** | 1.18 |
| 1-stearoyl-2-oleoyl-GPS (18:0/18:1) | Lipid | Phosphatidylserine (PS) | **1.99** | 1.09 |
| 1-(1-enyl-palmitoyl)-2-oleoyl-GPE (P-16:0/18:1) | Lipid | Plasmalogen | **1.73** | 1.08 |
| 1-(1-enyl-palmitoyl)-2-palmitoyl-GPC (P-16:0/16:0) | Lipid | Plasmalogen | **1.63** | 1.12 |
| 1-(1-enyl-palmitoyl)-2-palmitoleoyl-GPC (P-16:0/16:1) | Lipid | Plasmalogen | **1.33** | 1.14 |
| 1-(1-enyl-palmitoyl)-2-arachidonoyl-GPE (P-16:0/20:4) | Lipid | Plasmalogen | **1.49** | 1.16 |
| 1-(1-enyl-palmitoyl)-2-oleoyl-GPC (P-16:0/18:1) | Lipid | Plasmalogen | **1.37** | 1.1 |
| 1-(1-enyl-stearoyl)-2-oleoyl-GPE (P-18:0/18:1) | Lipid | Plasmalogen | **1.86** | 1.11 |
| 1-(1-enyl-palmitoyl)-2-linoleoyl-GPC (P-16:0/18:2) | Lipid | Plasmalogen | **1.53** | 1.17 |
| 1-(1-enyl-palmitoyl)-GPC (P-16:0) | Lipid | Lysoplasmalogen | **1.68** | 1.18 |
| 1-(1-enyl-oleoyl)-GPE (P-18:1) | Lipid | Lysoplasmalogen | **1.3** | 1.01 |
| 1-(1-enyl-stearoyl)-GPE (P-18:0) | Lipid | Lysoplasmalogen | **1.82** | 1.1 |
| 1-oleoyl-3-linoleoyl-glycerol (18:1/18:2) | Lipid | Diacylglycerol | **1.43** | 0.98 |
| 1-palmitoyl-3-linoleoyl-glycerol (16:0/18:2) | Lipid | Diacylglycerol | **1.44** | 1.12 |
| N-palmitoyl-sphinganine (d18:0/16:0) | Lipid | Sphingolipid metabolism | **1.86** | 1.14 |
| Stearoyl sphingomyelin (d18:1/18:0) | Lipid | Sphingolipid metabolism | **1.35** | 1.16 |
| N-palmitoyl-sphingosine (d18:1/16:0) | Lipid | Sphingolipid metabolism | **1.99** | 1.01 |
| Sphingomyelin (d18:1/20:1, d18:2/20:0) | Lipid | Sphingolipid metabolism | **1.22** | 0.85 |
| Behenoyl sphingomyelin (d18:1/22:0) | Lipid | Sphingolipid metabolism | **1.26** | 1.17 |
| Sphingomyelin (d18:1/21:0, d17:1/22:0, d16:1/23:0) | Lipid | Sphingolipid metabolism | **1.31** | 1.07 |
| Sphingomyelin (d18:1/17:0, d17:1/18:0, d19:1/16:0) | Lipid | Sphingolipid metabolism | **1.44** | 1.23 |
| Glycosyl-N-palmitoyl-sphingosine | Lipid | Sphingolipid metabolism | **2.07** | 1.05 |
| Lactosyl-N-palmitoyl-sphingosine | Lipid | Sphingolipid metabolism | **1.72** | 0.97 |
| 7-dehydrocholesterol | Lipid | Sterol | **2.07** | 0.98 |
| Cholate | Lipid | Primary bile acid metabolism | **1.53** | 1.16 |
| Glycochenodeoxycholate | Lipid | Primary bile acid metabolism | **1.67** | 1.04 |
| N6-carbamoylthreonyladenosine | Nucleotide | Purine metabolism, adenine-containing | **1.44** | 0.87 |
| Diadenosine triphosphate | Nucleotide | Purine metabolism, adenine-containing | **1.68** | 1.13 |
| Guanosine 5'-triphosphate | Nucleotide | Purine metabolism, guanine-containing | **4.7** | 1.51 |
| Uridine 5'-triphosphate (UTP) | Nucleotide | Pyrimidine metabolism, uracil-containing | **2.62** | 1.19 |
| Uridine 5'-diphosphate (UDP) | Nucleotide | Pyrimidine metabolism, uracil-containing | **1.77** | 1.35 |
| 2'-deoxyuridine | Nucleotide | Pyrimidine metabolism, uracil-containing | **2.43** | 0.71 |
| Cytidine triphosphate | Nucleotide | Pyrimidine metabolism, cytidine-containing | **4.08** | 1.24 |
| Cytosine | Nucleotide | Pyrimidine metabolism, cytidine-containing | **1.68** | 1.15 |
| Nicotinamide | Cofactors and vitamins | Nicotinate and nicotinamide metabolism | **1.8** | 0.91 |
| Nicotinamide riboside | Cofactors and vitamins | Nicotinate and nicotinamide metabolism | **1.32** | 0.99 |
| 1-methylnicotinamide | Cofactors and vitamins | Nicotinate and nicotinamide metabolism | **1.63** | 1 |
| Flavin mononucleotide (FMN) | Cofactors and vitamins | Riboflavin metabolism | **1.64** | 1.36 |
| Alpha-tocopherol | Cofactors and vitamins | Tocopherol metabolism | **1.44** | 1.01 |
| Pterin | Cofactors and vitamins | Pterin metabolism | **1.85** | 0.96 |
| Thiamine (Vitamin B1) | Cofactors and vitamins | Thiamine metabolism | **1.38** | 0.99 |
| Retinol (Vitamin A) | Cofactors and vitamins | Vitamin A metabolism | **1.48** | 1.04 |
| Pyridoxamine phosphate | Cofactors and vitamins | Vitamin B6 metabolism | **2.24** | 1 |
| Genistein | Xenobiotics | Food component/plant | **2.01** | 1.05 |
| Daidzein | Xenobiotics | Food component/plant | **1.65** | 1.09 |
| Pyrraline | Xenobiotics | Food component/plant | **1.53** | 1.03 |
| Phenol red | Xenobiotics | Chemical | **1.4** | 1.03 |
| Trizma acetate | Xenobiotics | Chemical | **1.58** | 0.99 |
| Glycine | Amino acid | Glycine, serine and threonine metabolism | **0.71** | 1.15 |
| N-acetylglycine | Amino acid | Glycine, serine and threonine metabolism | **0.59** | 0.91 |
| Sarcosine (N-methylglycine) | Amino acid | Glycine, serine and threonine metabolism | **0.39** | 1.21 |
| Dimethylglycine | Amino acid | Glycine, serine and threonine metabolism | **0.78** | 0.89 |
| Betaine | Amino acid | Glycine, serine and threonine metabolism | **0.8** | 1.01 |
| N-acetylserine | Amino acid | Glycine, serine and threonine metabolism | **0.36** | 0.85 |
| N-acetylthreonine | Amino acid | Glycine, serine and threonine metabolism | **0.33** | 1.08 |
| N-acetylalanine | Amino acid | Alanine and aspartate metabolism | **0.46** | 0.98 |
| N-acetylasparagine | Amino acid | Alanine and aspartate metabolism | **0.46** | 0.74 |
| N-acetylglutamine | Amino acid | Glutamate metabolism | **0.79** | 0.89 |
| Gamma-aminobutyrate (GABA) | Amino acid | Glutamate metabolism | **0.45** | 1.29 |
| 4-hydroxyglutamate | Amino acid | Glutamate metabolism | **0.58** | 1.02 |
| Glutamate, gamma-methyl ester | Amino acid | Glutamate metabolism | **0.27** | 1.12 |
| Pyroglutamine | Amino acid | Glutamate metabolism | **0.41** | 1.13 |
| N-methyl-4-aminobutyric acid | Amino acid | Glutamate metabolism | **0.23** | 1.23 |
| N-acetylhistidine | Amino acid | Histidine metabolism | **0.55** | 1.14 |
| Imidazole propionate | Amino acid | Histidine metabolism | **0.52** | 1.08 |
| Imidazole lactate | Amino acid | Histidine metabolism | **0.56** | 0.96 |
| 4-imidazoleacetate | Amino acid | Histidine metabolism | **0.73** | 1.01 |
| Cadaverine | Amino acid | Lysine metabolism | **0.4** | 1.04 |
| N-acetyl-cadaverine | Amino acid | Lysine metabolism | **0.61** | 0.98 |
| 5-aminovalerate | Amino acid | Lysine metabolism | **0.65** | 1.15 |
| N6-carboxyethyllysine | Amino acid | Lysine metabolism | **0.63** | 0.93 |
| Phenyllactate (PLA) | Amino acid | Phenylalanine and tyrosine metabolism | **0.68** | 1.3 |
| 3-(4-hydroxyphenyl)lactate | Amino acid | Phenylalanine and tyrosine metabolism | **0.72** | 1.05 |
| phenol sulfate | Amino acid | Phenylalanine and tyrosine metabolism | **0.41** | 0.97 |
| Beta-hydroxyisovaleroylcarnitine | Amino acid | Leucine, isoleucine and valine metabolism | **0.74** | 1.29 |
| Alpha-hydroxyisovalerate | Amino acid | Leucine, isoleucine and valine metabolism | **0.68** | 0.82 |
| 2-methylbutyrylcarnitine (C5) | Amino acid | Leucine, isoleucine and valine metabolism | **0.6** | 0.98 |
| Tiglylcarnitine | Amino acid | Leucine, isoleucine and valine metabolism | **0.61** | 1.2 |
| Ethylmalonate | Amino acid | Leucine, isoleucine and valine metabolism | **0.38** | 0.99 |
| N-acetylvaline | Amino acid | Leucine, isoleucine and valine metabolism | **0.47** | 1.01 |
| Isobutyrylcarnitine | Amino acid | Leucine, isoleucine and valine metabolism | **0.59** | 1.02 |
| Alpha-hydroxyisocaproate | Amino acid | Leucine, isoleucine and valine metabolism | **0.35** | 0.79 |
| N-acetylmethionine | Amino acid | Methionine, cysteine, SAM and taurine metabolism | **0.73** | 0.98 |
| N-formylmethionine | Amino acid | Methionine, cysteine, SAM and taurine metabolism | **0.69** | 1.02 |
| S-adenosylhomocysteine (SAH) | Amino acid | Methionine, cysteine, SAM and taurine metabolism | **0.8** | 1.07 |
| Hypotaurine | Amino acid | Methionine, cysteine, SAM and taurine metabolism | **0.48** | 1.13 |
| Taurine | Amino acid | Methionine, cysteine, SAM and taurine metabolism | **0.48** | 1.06 |
| N-acetyltaurine | Amino acid | Methionine, cysteine, SAM and taurine metabolism | **0.19** | 0.92 |
| Creatine | Amino acid | Creatine metabolism | **0.71** | 1.07 |
| 4-acetamidobutanoate | Amino acid | Polyamine metabolism | **0.75** | 0.96 |
| 4-guanidinobutanoate | Amino acid | Guanidino and acetamido metabolism | **0.42** | 1.13 |
| Ophthalmate | Amino acid | Glutathione metabolism | **0.42** | 1.04 |
| Gamma-glutamylglutamine | Peptide | Gamma-glutamyl amino acid | **0.75** | 1.14 |
| Gamma-glutamylglycine | Peptide | Gamma-glutamyl amino acid | **0.34** | 1.3 |
| Gamma-glutamylthreonine | Peptide | Gamma-glutamyl amino acid | **0.51** | 1.02 |
| Gamma-glutamyl-2-aminobutyrate | Peptide | Gamma-glutamyl amino acid | **0.75** | 1.11 |
| Ribonate | Carbohydrate | Pentose metabolism | **0.45** | 0.96 |
| Galactonate | Carbohydrate | Fructose, mannose and galactose metabolism | **0.38** | 0.71 |
| Erythronate | Carbohydrate | Aminosugar metabolism | **0.34** | 0.97 |
| Succinate | Energy | TCA cycle | **0.57** | 0.83 |
| Fumarate | Energy | TCA cycle | **0.8** | 0.9 |
| Myristoleate (14:1n5) | Lipid | Long-chain fatty acid | **0.56** | 0.9 |
| Palmitoleate (16:1n7) | Lipid | Long-chain fatty acid | **0.59** | 0.82 |
| 2-hydroxyglutarate | Lipid | Fatty acid, dicarboxylate | **0.6** | 0.86 |
| Butyrylcarnitine | Lipid | Fatty acid metabolism (also BCAA metabolism) | **0.58** | 1.05 |
| Propionylcarnitine | Lipid | Fatty acid metabolism (also BCAA metabolism) | **0.58** | 1.12 |
| N-palmitoyl glycine | Lipid | Fatty acid metabolism (acyl glycine) | **0.54** | 1 |
| 3-hydroxybutyrylcarnitine (1) | Lipid | Fatty acid metabolism (acyl carnitine) | **0.66** | 1.3 |
| 3-hydroxybutyrylcarnitine (2) | Lipid | Fatty acid metabolism (acyl carnitine) | **0.55** | 1.17 |
| Hexanoylcarnitine | Lipid | Fatty acid metabolism (acyl carnitine) | **0.49** | 1.03 |
| Octanoylcarnitine | Lipid | Fatty acid metabolism (acyl carnitine) | **0.64** | 1 |
| Decanoylcarnitine | Lipid | Fatty acid metabolism (acyl carnitine) | **0.72** | 1.21 |
| Myristoylcarnitine | Lipid | Fatty acid metabolism (acyl carnitine) | **0.69** | **1.39** |
| Palmitoylcarnitine | Lipid | Fatty acid metabolism (acyl carnitine) | **0.72** | 1.2 |
| Linoleoylcarnitine | Lipid | Fatty acid metabolism (acyl carnitine) | **0.52** | 1.33 |
| Oleoylcarnitine | Lipid | Fatty acid metabolism (acyl carnitine) | **0.68** | 1.21 |
| Myristoleoylcarnitine | Lipid | Fatty acid metabolism (acyl carnitine) | **0.63** | **1.45** |
| Deoxycarnitine | Lipid | Carnitine metabolism | **0.64** | 1.14 |
| 4-hydroxybutyrate (GHB) | Lipid | Fatty acid, monohydroxy | **0.51** | 0.86 |
| 2-hydroxypalmitate | Lipid | Fatty acid, monohydroxy | **0.58** | 0.99 |
| 1-palmitoleoyl-2-oleoyl-GPC (16:1/18:1) | Lipid | Phospholipid metabolism | **0.59** | 0.85 |
| 1,2-dipalmitoleoyl-GPE (16:1/16:1) | Lipid | Phospholipid metabolism | **0.51** | 1.11 |
| 1-palmitoyl-GPS (16:0) | Lipid | Lysolipid | **0.47** | 0.93 |
| Glycerol | Lipid | Glycerolipid metabolism | **0.7** | 0.83 |
| Glycerophosphoglycerol | Lipid | Glycerolipid metabolism | **0.46** | 0.93 |
| 1-oleoylglycerol (18:1) | Lipid | Monoacylglycerol | **0.64** | 0.88 |
| 1-palmitoleoyl-2-oleoyl-glycerol (16:1/18:1) | Lipid | Diacylglycerol | **0.68** | 1.28 |
| 1-palmitoleoyl-3-oleoyl-glycerol (16:1/18:1) | Lipid | Diacylglycerol | **0.52** | 0.95 |
| Sphinganine | Lipid | Sphingolipid metabolism | **0.46** | 0.84 |
| Phytosphingosine | Lipid | Sphingolipid metabolism | **0.38** | 0.85 |
| Sphingosine | Lipid | Sphingolipid metabolism | **0.55** | 0.91 |
| Sphingomyelin (d18:2/14:0, d18:1/14:1) | Lipid | Sphingolipid metabolism | **0.7** | 0.83 |
| Sphingomyelin (d18:2/16:0, d18:1/16:1) | Lipid | Sphingolipid metabolism | **0.7** | 0.85 |
| Inosine | Nucleotide | Purine metabolism, (hypo)xanthine/inosine-containing | **0.55** | 1.09 |
| Hypoxanthine | Nucleotide | Purine metabolism, (hypo)xanthine/inosine-containing | **0.53** | 0.8 |
| Xanthine | Nucleotide | Purine metabolism, (hypo)xanthine/inosine-containing | **0.59** | 0.9 |
| Allantoic acid | Nucleotide | Purine metabolism, (hypo)xanthine/inosine-containing | **0.79** | 1.03 |
| N6-succinyladenosine | Nucleotide | Purine metabolism, adenine-containing | **0.48** | 0.86 |
| Guanosine | Nucleotide | Purine metabolism, guanine-containing | **0.64** | 1.18 |
| Orotidine | Nucleotide | Pyrimidine metabolism, orotate-containing | **0.1** | 0.92 |
| Uridine | Nucleotide | Pyrimidine metabolism, uracil-containing | **0.72** | 0.86 |
| Uracil | Nucleotide | Pyrimidine metabolism, uracil-containing | **0.37** | 0.78 |
| Beta-alanine | Nucleotide | Pyrimidine metabolism, uracil-containing | **0.5** | 1.04 |
| 5-methylcytidine | Nucleotide | Pyrimidine metabolism, cytidine-containing | **0.82** | 1 |
| 2'-deoxycytidine | Nucleotide | Pyrimidine metabolism, cytidine-containing | **0.78** | 1 |
| 3-aminoisobutyrate | Nucleotide | Pyrimidine metabolism, thymine-containing | **0.32** | 1.06 |
| Nicotinamide adenine dinucleotide reduced (NADH) | Cofactors and vitamins | Nicotinate and nicotinamide metabolism | **0.56** | 1.08 |
| Trigonelline (N'-methylnicotinate) | Cofactors and vitamins | Nicotinate and nicotinamide metabolism | **0.58** | 0.99 |
| Adenosine 5'-diphosphoribose (ADP-ribose) | Cofactors and vitamins | Nicotinate and nicotinamide metabolism | **0.72** | **1.53** |
| Pantothenate | Cofactors and vitamins | Pantothenate and CoA metabolism | **0.8** | 0.98 |
| Gulonic acid | Cofactors and vitamins | Ascorbate and aldarate metabolism | **0.33** | 0.94 |
| 5-methyltetrahydrofolate (5MeTHF) | Cofactors and vitamins | Folate metabolism | **0.58** | 0.98 |
| Pyridoxine (Vitamin B6) | Cofactors and vitamins | Vitamin B6 metabolism | **0.31** | 1 |
| O-methylcatechol sulfate | Xenobiotics | Benzoate metabolism | **0.76** | 1.05 |
| 4-vinylphenol sulfate | Xenobiotics | Benzoate metabolism | **0.62** | 1.15 |
| Beta-guanidinopropanoate | Xenobiotics | Food component/plant | **0.36** | 1.21 |
| Ergothioneine | Xenobiotics | Food component/plant | **0.81** | **1.37** |
| Erythritol | Xenobiotics | Food component/plant | **0.83** | 1.01 |
| Thymol sulfate | Xenobiotics | Food component/plant | **0.27** | 1.15 |
| Daidzein sulfate (2) | Xenobiotics | Food component/plant | **0.37** | 1.1 |
| S-carboxymethyl-L-cysteine | Xenobiotics | Drug | **0.68** | 1.05 |
| 2-aminophenol sulfate | Xenobiotics | Chemical | **0.24** | 1.12 |
| Benzoylcarnitine | Xenobiotics | Chemical | **0.39** | 1.24 |
