# Supplementary material for: Cytidine deaminase deficiency in tumor cells is associated with sensitivity to a naphthol derivative and a decrease in oncometabolite levels
Source: Cell Mol Life Sci. 2022 Aug 4;79(8):465. doi: 10.1007/s00018-022-04487-9 (PMC9352748; doi:10.1007/s00018-022-04487-9)
Supplement: Supplementary file 4 — Supplementary file4 (DOCX 43 KB) [file 18_2022_4487_MOESM4_ESM.docx]

Supplementary table 4

| Metabolite name | Super pathway | Sub pathway | HeLa-shCDA HeLa-Ctrl | HeLa-shCDA + X55 HeLa-shCDA |
| --- | --- | --- | --- | --- |
| N-acetylserine | Amino acid | Glycine, serine and threonine metabolism | **0.61** | **0.36** |
| N-acetylasparagine | Amino acid | Alanine and aspartate metabolism | **0.52** | **0.46** |
| Creatine | Amino acid | Creatine metabolism | **0.46** | **0.71** |
| Gamma-glutamylglutamine | Peptide | Gamma-glutamyl amino acid | **0.2** | **0.75** |
| Succinate | Energy | TCA cycle | **0.64** | **0.57** |
| Fumarate | Energy | TCA cycle | **0.65** | **0.8** |
| 2-methylcitrate/homocitrate | Energy | TCA cycle | **0.54** | **0.66** |
| Myristoleate (14:1n5) | Lipid | Long chain fatty acid | **0.76** | **0.56** |
| Palmitoleate (16:1n7) | Lipid | Long chain fatty acid | **0.6** | **0.59** |
| 2-hydroxyglutarate | Lipid | Fatty acid, dicarboxylate | **0.65** | **0.6** |
| Glycerol | Lipid | Glycerolipid metabolism | **0.71** | **0.7** |
| 1-palmitoleoyl-3-oleoyl-glycerol (16:1/18:1) | Lipid | Diacylglycerol | **0.77** | **0.52** |
| Inosine | Nucleotide | Purine metabolism, (hypo)xanthine/inosine-containing | **0.27** | **0.55** |
| Hypoxanthine | Nucleotide | Purine metabolism, (hypo)xanthine/inosine-containing | **0.31** | **0.53** |
| Xanthine | Nucleotide | Purine metabolism, (hypo)xanthine/inosine-containing | **0.39** | **0.59** |
| N6-succinyladenosine | Nucleotide | Purine metabolism, adenine-containing | **0.08** | **0.48** |
| Guanosine | Nucleotide | Purine metabolism, guanine-containing | **0.25** | **0.64** |
| Uridine | Nucleotide | Pyrimidine metabolism, uracil-containing | **0.29** | **0.72** |
| Uracil | Nucleotide | Pyrimidine metabolism, uracil-containing | **0.31** | **0.37** |
| 5-methyltetrahydrofolate (5MeTHF) | Cofactors and vitamins | Folate metabolism | **0.65** | **0.58** |
| S-carboxymethyl-L-cysteine | Xenobiotics | Drug | **0.59** | **0.68** |
| Asparagine | Amino acid | Alanine and aspartate metabolism | **1.3** | **1.53** |
| Histidine | Amino acid | Histidine metabolism | **1.77** | **1.52** |
| 1-methylhistidine | Amino acid | Histidine metabolism | **2.22** | **1.52** |
| 3-methylhistidine | Amino acid | Histidine metabolism | **1.66** | **1.75** |
| N6,N6,N6-trimethyllysine | Amino acid | Lysine metabolism | **1.47** | **1.71** |
| Phenylalanine | Amino acid | Phenylalanine and tyrosine metabolism | **1.5** | **1.48** |
| Tyrosine | Amino acid | Phenylalanine and tyrosine metabolism | **1.59** | **1.52** |
| Tryptophan | Amino acid | Tryptophan metabolism | **1.74** | **1.52** |
| 5-hydroxyindoleacetate | Amino acid | Tryptophan metabolism | **1.58** | **2.37** |
| Leucine | Amino acid | Leucine, isoleucine and valine metabolism | **1.53** | **1.48** |
| 5-methylnorleucine | Amino acid | Leucine, isoleucine and valine metabolism | **1.92** | **1.53** |
| Isoleucine | Amino acid | Leucine, isoleucine and valine metabolism | **1.5** | **1.47** |
| Valine | Amino acid | Leucine, isoleucine and valine metabolism | **1.68** | **1.5** |
| Methionine | Amino acid | Methionine, cysteine, SAM and taurine metabolism | **1.6** | **1.48** |
| Methionine sulfone | Amino acid | Methionine, cysteine, SAM and taurine metabolism | **2.24** | **1.6** |
| Methionine sulfoxide | Amino acid | Methionine, cysteine, SAM and taurine metabolism | **2.25** | **1.55** |
| S-adenosylmethionine (SAM) | Amino acid | Methionine, cysteine, SAM and taurine metabolism | **1.29** | **1.35** |
| Ornithine | Amino acid | Urea cycle, arginine and proline metabolism | **1.46** | **1.91** |
| Citrulline | Amino acid | Urea cycle, arginine and proline metabolism | **3.3** | **1.67** |
| Homoarginine | Amino acid | Urea cycle, arginine and proline metabolism | **1.57** | **1.74** |
| Homocitrulline | Amino acid | Urea cycle, arginine and proline metabolism | **2.07** | **1.74** |
| N-delta-acetylornithine | Amino acid | Urea cycle, arginine and proline metabolism | **1.93** | **1.59** |
| Guanidinoacetate | Amino acid | Creatine metabolism | **2.32** | **3.91** |
| Spermine | Amino acid | Polyamine metabolism | **2.72** | **1.86** |
| Spermidine | Amino acid | Polyamine metabolism | **1.7** | **1.82** |
| 5-methylthioadenosine (MTA) | Amino acid | Polyamine metabolism | **1.44** | **1.36** |
| Glycylleucine | Peptide | Dipeptide | **1.31** | **1.27** |
| UDP-glucose | Carbohydrate | Nucleotide sugar | **1.3** | **1.52** |
| UDP-galactose | Carbohydrate | Nucleotide sugar | **1.35** | **1.53** |
| N-palmitoyltaurine | Lipid | Endocannabinoid | **1.94** | **1.89** |
| Inositol 1-phosphate (I1P) | Lipid | Inositol metabolism | **6.07** | **1.73** |
| Choline phosphate | Lipid | Phospholipid metabolism | **1.55** | **1.39** |
| 1-palmitoyl-2-stearoyl-GPC (16:0/18:0) | Lipid | Phospholipid metabolism | **1.54** | **1.39** |
| 1,2-dioleoyl-GPS (18:1/18:1) | Lipid | Phospholipid metabolism | **1.46** | **1.36** |
| 1-stearoyl-2-arachidonoyl-GPS (18:0/20:4) | Lipid | Phosphatidylserine (PS) | **1.91** | **1.68** |
| 1-stearoyl-GPS (18:0) | Lipid | Lysolipid | **1.33** | **1.29** |
| 1-(1-enyl-stearoyl)-2-arachidonoyl-GPE (P-18:0/20:4) | Lipid | Plasmalogen | **1.45** | **1.8** |
| 1-(1-enyl-stearoyl)-GPE (P-18:0) | Lipid | Lysoplasmalogen | **1.34** | **1.82** |
| N-palmitoyl-sphinganine (d18:0/16:0) | Lipid | Sphingolipid metabolism | **1.49** | **1.86** |
| Glycosyl-N-stearoyl-sphingosine | Lipid | Sphingolipid metabolism | **1.23** | **2.32** |
| Glycosyl-N-palmitoyl-sphingosine | Lipid | Sphingolipid metabolism | **1.49** | **2.07** |
| Lactosyl-N-palmitoyl-sphingosine | Lipid | Sphingolipid metabolism | **3.43** | **1.72** |
| Cholate | Lipid | Primary bile acid metabolism | **1.51** | **1.53** |
| Glycochenodeoxycholate | Lipid | Primary bile acid metabolism | **1.55** | **1.67** |
| Adenosine 5'-diphosphate (ADP) | Nucleotide | Purine metabolism, adenine-containing | **6.08** | **1.54** |
| 2'-deoxyadenosine 5'-monophosphate | Nucleotide | Purine metabolism, adenine-containing | **3.36** | **2.49** |
| Guanosine 5'-triphosphate | Nucleotide | Purine metabolism, guanine-containing | **9.27** | **4.7** |
| Uridine 5'-triphosphate (UTP) | Nucleotide | Pyrimidine metabolism, uracil-containing | **2.93** | **2.62** |
| Uridine 5'-diphosphate (UDP) | Nucleotide | Pyrimidine metabolism, uracil-containing | **2.82** | **1.77** |
| Cytidine triphosphate | Nucleotide | Pyrimidine metabolism, cytidine-containing | **3.13** | **4.08** |
| Cytidine diphosphate | Nucleotide | Pyrimidine metabolism, cytidine-containing | **3.45** | **2.85** |
| 1-methylnicotinamide | Cofactors and vitamins | Nicotinate and nicotinamide metabolism | **2.21** | **1.63** |
| Pyrraline | Xenobiotics | Food component/plant | **2.39** | **1.53** |
| Phenol red | Xenobiotics | Chemical | **1.38** | **1.4** |
| Trizma acetate | Xenobiotics | Chemical | **1.31** | **1.58** |
| **N-acetylaspartate (NAA)** | Amino acid | Alanine and aspartate metabolism | **0.76** | **1.31** |
| **Cystathionine** | Amino acid | Methionine, cysteine, SAM and taurine metabolism | **0.67** | **3.97** |
| **Cysteine** | Amino acid | Methionine, cysteine, SAM and taurine metabolism | **0.48** | **1.47** |
| **Cysteine-glutathione disulfide** | Amino acid | Glutathione metabolism | **0.38** | **1.83** |
| **5-oxoproline** | Amino acid | Glutathione metabolism | **0.71** | **1.45** |
| **Fructose-6-phosphate** | Carbohydrate | Glycolysis, gluconeogenesis, and pyruvate metabolism | **0.6** | **3.34** |
| **Choline** | Lipid | Phospholipid metabolism | **0.52** | **1.32** |
| **Glycerophosphorylcholine (GPC)** | Lipid | Phospholipid metabolism | **0.42** | **2.07** |
| **1-stearoyl-2-oleoyl-GPE (18:0/18:1)** | Lipid | Phospholipid metabolism | **0.7** | **1.41** |
| **1-oleoyl-3-linoleoyl-glycerol (18:1/18:2)** | Lipid | Diacylglycerol | **0.76** | **1.43** |
| **7-dehydrocholesterol** | Lipid | Sterol | **0.3** | **2.07** |
| **N6-carbamoylthreonyladenosine** | Nucleotide | Purine metabolism, adenine-containing | **0.58** | **1.44** |
| **T2'-deoxyuridine** | Nucleotide | Pyrimidine metabolism, uracil-containing | **0.04** | **2.43** |
| **Thymidine 5'-monophosphate** | Nucleotide | Pyrimidine metabolism, thymine-containing | **0.66** | **1.55** |
| **Pterin** | Cofactors and vitamins | Pterin metabolism | **0.76** | **1.85** |
| **Genistein** | Xenobiotics | Food component/plant | **0.61** | **2.01** |
| **Glutamate, gamma-methyl ester** | Amino acid | Glutamate metabolism | **1.96** | **0.27** |
| **N-acetylhistidine** | Amino acid | Histidine metabolism | **1.48** | **0.55** |
| **Imidazole lactate** | Amino acid | Histidine metabolism | **1.91** | **0.56** |
| **Cadaverine** | Amino acid | Lysine metabolism | **1.79** | **0.4** |
| **N-acetyl-cadaverine** | Amino acid | Lysine metabolism | **1.6** | **0.61** |
| **Phenyllactate (PLA)** | Amino acid | Phenylalanine and tyrosine metabolism | **2.55** | **0.68** |
| **3-(4-hydroxyphenyl)lactate** | Amino acid | Phenylalanine and tyrosine metabolism | **2.71** | **0.72** |
| **beta-hydroxyisovaleroylcarnitine** | Amino acid | Leucine, isoleucine and valine metabolism | **1.41** | **0.74** |
| **2-methylbutyrylcarnitine (C5)** | Amino acid | Leucine, isoleucine and valine metabolism | **1.76** | **0.6** |
| **Alpha-hydroxyisocaproate** | Amino acid | Leucine, isoleucine and valine metabolism | **5.18** | **0.35** |
| **S-adenosylhomocysteine (SAH)** | Amino acid | Methionine, cysteine, SAM and taurine metabolism | **1.24** | **0.8** |
| **Ophthalmate** | Amino acid | Glutathione metabolism | **26** | **0.42** |
| **Gamma-glutamylglycine** | Peptide | Gamma-glutamyl amino acid | **1.76** | **0.34** |
| **Gamma-glutamylthreonine** | Peptide | Gamma-glutamyl amino acid | **14.75** | **0.51** |
| **Butyrylcarnitine** | Lipid | Fatty acid metabolism (also BCAA metabolism) | **1.63** | **0.58** |
| **Hexanoylcarnitine** | Lipid | Fatty acid metabolism (acyl carnitine) | **1.5** | **0.49** |
| **Octanoylcarnitine** | Lipid | Fatty acid metabolism (acyl carnitine) | **2.25** | **0.64** |
| **Decanoylcarnitine** | Lipid | Fatty acid metabolism (acyl carnitine) | **1.69** | **0.72** |
| **Myristoylcarnitine** | Lipid | Fatty acid metabolism (acyl carnitine) | **1.87** | **0.69** |
| **Palmitoylcarnitine** | Lipid | Fatty acid metabolism (acyl carnitine) | **1.44** | **0.72** |
| **Myristoleoylcarnitine** | Lipid | Fatty acid metabolism (acyl carnitine) | **1.51** | **0.63** |
| **Deoxycarnitine** | Lipid | Carnitine metabolism | **1.48** | **0.64** |
| **1-palmitoyl-GPS (16:0)** | Lipid | Lysolipid | **2.27** | **0.47** |
| **Sphingomyelin (d18:2/16:0, d18:1/16:1)** | Lipid | Sphingolipid metabolism | **1.62** | **0.7** |
| **5-methylcytidine** | Nucleotide | Pyrimidine metabolism, cytidine-containing | **2.94** | **0.82** |
| **2'-deoxycytidine** | Nucleotide | Pyrimidine metabolism, cytidine-containing | **1.71** | **0.78** |
| **Adenosine 5'-diphosphoribose (ADP-ribose)** | Cofactors and vitamins | Nicotinate and nicotinamide metabolism | **1.43** | **0.72** |
| **Pyridoxine (Vitamin B6)** | Cofactors and vitamins | Vitamin B6 metabolism | **7.67** | **0.31** |
| **4-vinylphenol sulfate** | Xenobiotics | Benzoate metabolism | **1.51** | **0.62** |
| **Ergothioneine** | Xenobiotics | Food component/plant | **1.36** | **0.81** |
| **Thymol sulfate** | Xenobiotics | Food component/plant | **3.13** | **0.27** |
| **Daidzein sulfate (2)** | Xenobiotics | Food component/plant | **4.06** | **0.37** |
| **2-aminophenol sulfate** | Xenobiotics | Chemical | **1.49** | **0.24** |
