# Supplementary material for: Cytidine deaminase deficiency in tumor cells is associated with sensitivity to a naphthol derivative and a decrease in oncometabolite levels
Source: Cell Mol Life Sci. 2022 Aug 4;79(8):465. doi: 10.1007/s00018-022-04487-9 (PMC9352748; doi:10.1007/s00018-022-04487-9)
Supplement: Supplementary file 5 — Supplementary file5 (PDF 964 KB) [file 18_2022_4487_MOESM5_ESM.pdf]

Supplementary Information

Cytidine deaminase deficiency in tumor cells is associated with sensitivity to a naphthol derivative and a decrease in oncometabolite levels

Hamza Mameri[1,2,3,8*], Géraldine Buhagiar-Labarchède[1,2,3*], Gaëlle Fontaine[1,2,3], Céline Corcelle[4,5,6], Caroline Barette[7], Rosine Onclercq-Delic[1,2,3], Claire Beauvineau[4,5,6], Florence Mahuteau-Betzer[4,5,6#] and Mounira Amor-Guéret[1,2,3#]

Authors' affiliations:

[1]Institut Curie, PSL Research University, CNRS UMR 3348, F-91405, Orsay, France.

[2]CNRS UMR 3348, Centre Universitaire, Bât. 110. 91405, Orsay, France

[3]Université Paris-Saclay, CNRS UMR 3348, F-91405 Orsay, France.

[4]Institut Curie, PSL Research University, CNRS UMR 9187, INSERM U1196, F-91405, Orsay, France.

[5] CNRS UMR 9187, INSERM, U1196, Centre Universitaire, Bât. 110. 91405, Orsay, France

[6] Université Paris-Saclay, CNRS, UMR 9187, INSERM, U1196, F-91405 Orsay, France.

[7] CEA/IRIG/Gen & Chem, Univ. Grenoble Alpes, 38000 Grenoble, France.

[8] Present address: INRAE, Montpellier University, Montpellier SupAgro, UMR 1208 IATE, F-34060 Montpellier, France

[*]Hamza Mameri and Géraldine Buhagiar-Labarchède contributed as co-first authors.

#Co-corresponding authors:

Mounira Amor-Guéret, Institut Curie, CNRS UMR 3348, Centre Universitaire, Bât. 110. 91405, Orsay, France. Phone: 33 1 69 86 30 53; E-mail: **mounira.amor@curie.fr**

Florence Mahuteau-Betzer, Institut Curie, CNRS UMR 9187, INSERM U1196, Centre Universitaire, Bât. 110. 91405, Orsay, France. Phone: 33 1 69 86 7159; E-mail: **florence.mahuteau@curie.fr**

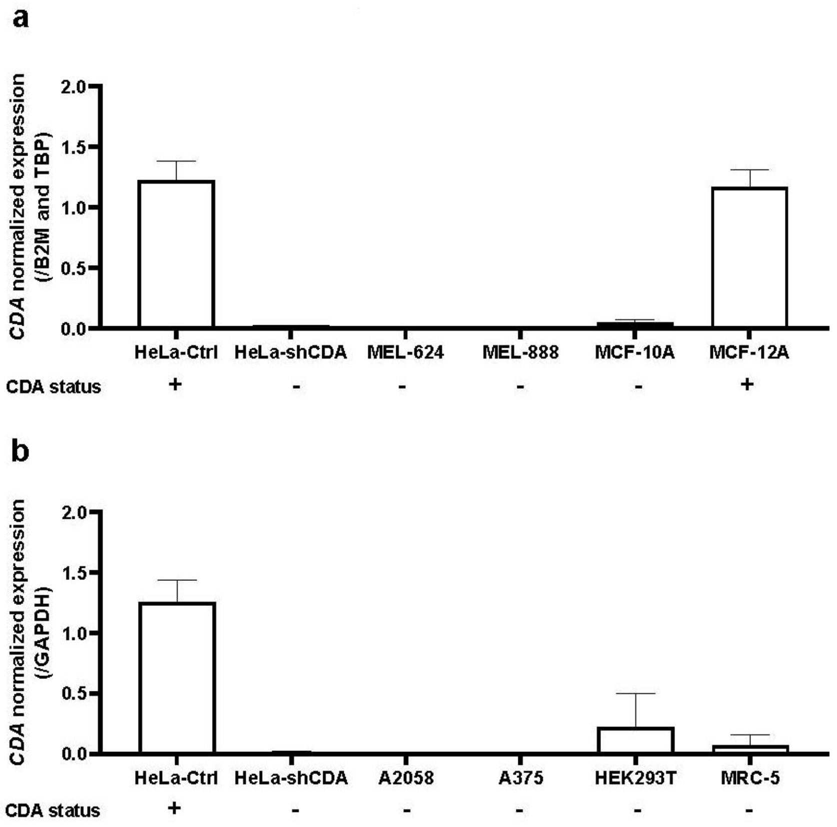

Supplementary Figure 1. CDA expression status of the cell lines not presented by Mameri et al. (2017) [1]. (**a**) *CDA* levels were monitored by RT-qPCR in HeLa-Ctrl and HeLa-shCDA (as controls) and in MEL-624, MEL-888, MCF10A and MCF-12A cells. *B2M* and *TBP* were used as housekeeping genes for normalization. The error bars represent means ± SD of three independent experiments (except for MCF12A, 2 independent experiments). (**b**) *CDA* levels were monitored by RT-qPCR in HeLa-Ctrl and HeLa-shCDA (as controls) and in A2058, A375, HEK293T and MRC5 cells. *GAPDH* was used as housekeeping gene for normalization. The error bars represent means ± SD for of three independent experiments (except for A2058 and HEK293T, 2 independent experiments).

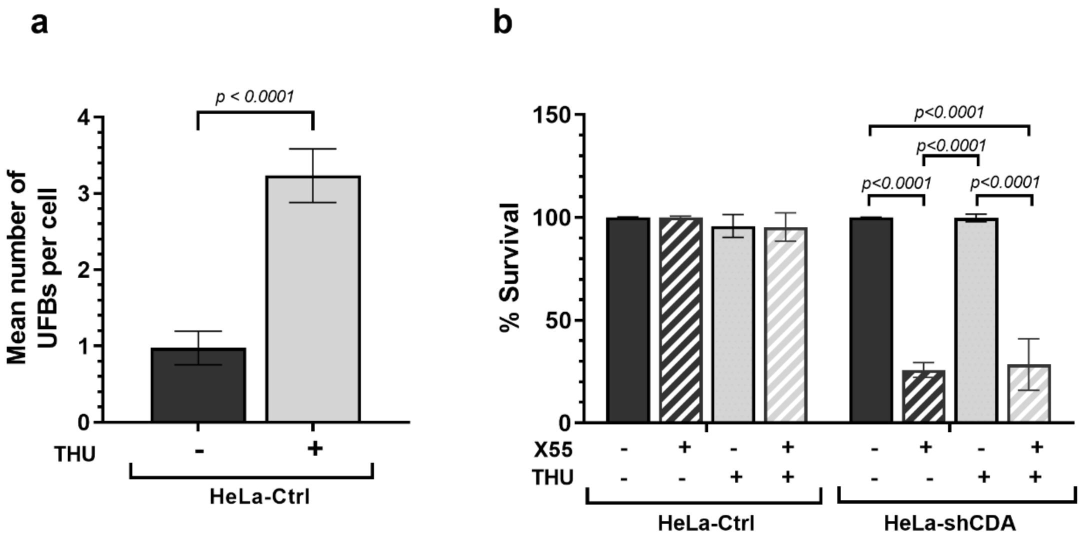

Supplementary Figure 2. CDA activity is dispensable for X55 cytotoxicity in CDA-depleted HeLa cells. **a** Mean number of UFBs per cell in HeLa control cells (HeLa-Ctrl) left untreated (black bars) or treated for 96 h (2x48 h) with 100 µM THU (gray bars). Error bars represent means ± SD for two independent experiments. The significance of differences was assessed in Mann-Whitney tests. $P < 0.05$ was considered statistically significant. **b** HeLa control cells (HeLa-Ctrl) and CDA-depleted HeLa cells (HeLa-shCDA) were left untreated (black bars) or were subjected to pretreatment for 96 h (2x48 h) with 100 µM THU (gray bars). They were then left untreated or were treated with 1 µM X55 for 24 h (hatched bars). Error bars represent means ± SD for three independent experiments. The significance of differences was assessed by two-way ANOVA. $P < 0.05$ was considered statistically significant.

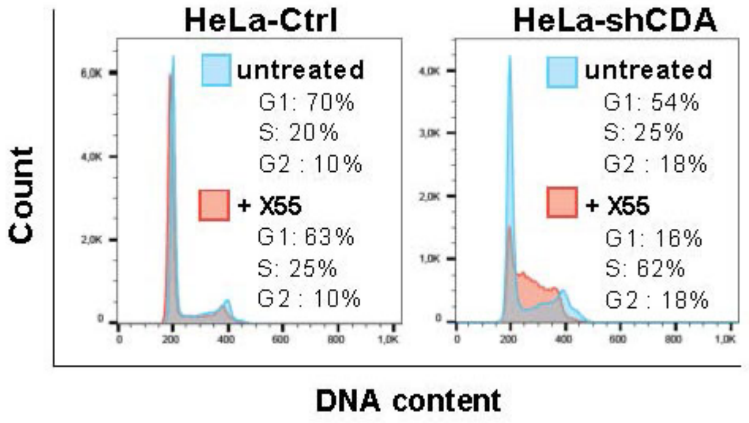

Supplementary Figure 3. Representative cell cycle distribution for HeLa-Ctrl cells (left panel) and CDA-depleted HeLa cells (right panel), left untreated (blue) or treated for 24 hours with 1 µM X55 (red). The percentages of cells in the G1, S and G2 phases are indicated.

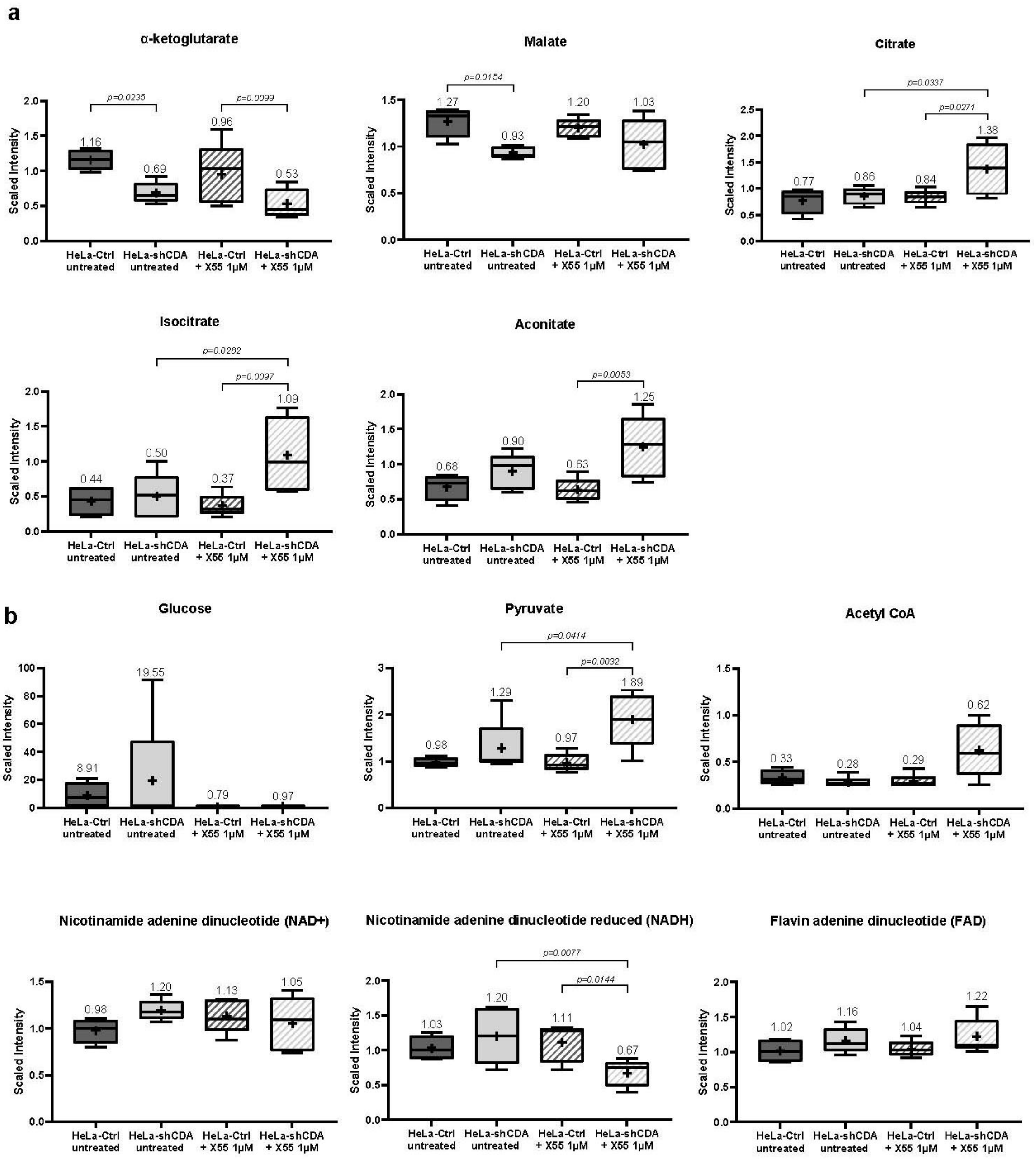

Supplementary Figure 4. Abundance of TCA cycle metabolites and key players of this pathway in CDA-depleted HeLa cells and in control HeLa cells. Measurement of the levels of citrate, isocitrate, aconinate, α-ketoglutarate, and malate **(a)** and of glucose, pyruvate, acetyl-CoA, NAD^+, NADH, and FAD **(b)** in HeLa-Ctrl cells (dark gray) and CDA-depleted HeLa cells (light gray) left untreated (block-shaded) or treated for 24 hours with 1 µM X55 (hatched). The error bars represent means ± SD for four or five independent experiments. The significance of

differences was assessed by two-way ANOVA. $P < 0.05$ was considered statistically significant.

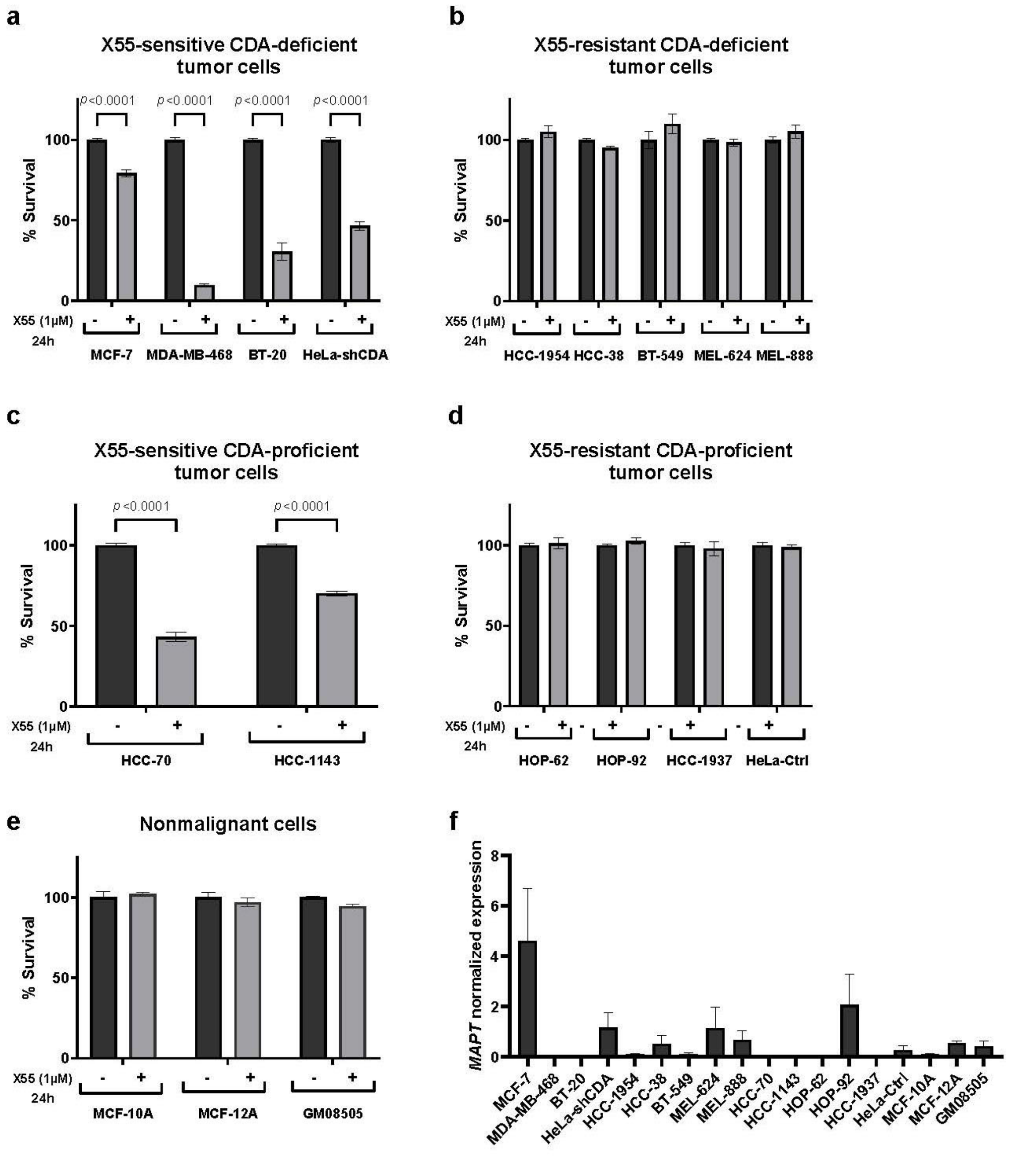

Supplementary Figure 5. X55 sensitivity of CDA-deficient cells and CDA-proficient cells. **a, b, c, d, e** X55-sensitive CDA-deficient cells (**a**), X55-resistant CDA-deficient cells (**b**), X55-sensitive CDA-proficient cells (**c**), X55-resistant CDA-proficient cells (**d**), nonmalignant cells (**e**), left untreated (black bars) or treated with 1 μM X55 for 24 h (gray bars) and the percentage

of cells surviving. The significance of differences was assessed by two-way ANOVA. The error bars represent means ±SD for at least three independent experiments. $P < 0.05$ was considered statistically significant. **f** Relative basal *MAPT* mRNA levels in all cell lines tested. *MAPT* expression levels were monitored by RT-qPCR. *B2M* and *TBP* were used as housekeeping genes for normalization. The error bars represent means ± SD for at least two independent experiments.

Supplementary Table 1. Raw metabolomic data. Values are normalized in terms of raw area counts (Metabolon Inc.). The shaded column corresponds to the sample from the control HeLa group (without X55 treatment) that was considered to be an outlier and was excluded from the statistical analysis. ***Provided as a separate file (.xlsx)***

Supplementary Table 2. List of metabolites for which levels were significantly deregulated in CDA-depleted HeLa cells relative to control HeLa cells. The significance of differences in metabolite levels was assessed by ANOVA ($P < 0.05$). Downregulated metabolites are shown in green, and upregulated metabolites are shown in red.

Provided as a separate file (.doc)

Supplementary Table 3. List of the metabolites with levels significantly deregulated by X55 treatment in control HeLa cells (49) and CDA-depleted HeLa cells (270). The significance of differences in metabolite levels was assessed by ANOVA ($P < 0.05$). Upregulated metabolites are shown in red, and downregulated metabolites are shown in green. The names of the 17 metabolites deregulated in both cells in response to X55 are indicated in bold.

Provided as a separate file (.doc)

Supplementary Table 4. List of 125 metabolites with levels significantly deregulated in both untreated CDA-depleted HeLa cells and in X55-treated CDA-depleted HeLa cells relative to untreated HeLa-Ctrl or HeLa-shCDA cells, respectively. The significance of differences in metabolite levels was assessed by ANOVA ($P < 0.05$). The metabolites upregulated in both sets of conditions (55) are shown in red, and those downregulated in both sets of conditions (21) are shown in green. The metabolites deregulated in opposite ways (49) are shown in bold.

Provided as a separate file (.doc)

Supplementary Material 1. Growth conditions for the cell lines used in this study

	Cell line	Medium
Cervical cancer	HeLa-Ctrl	DMEM + 1% glutamine + 1% penicillin/streptomycin + 10% FBS
Cervical cancer	HeLa-shCDA	DMEM + 1% glutamine + 1% penicillin/streptomycin + 10% FBS
Breast cancer	MCF-7	DMEM + 1% glutamine + 1% penicillin/streptomycin + 10% FBS
Breast cancer	BT-20	MEM (Eagle) +1% glutamine+ 1% penicillin/streptomycin + 10% FBS + 1.5 g/L sodium bicarbonate + 0.1 mM non-essential amino acids + 1 mM sodium pyruvate
Breast cancer	MDA-MB-468	RPMI-1640 + 1% glutamine + 1% penicillin/streptomycin + 10% FBS
Breast cancer	ZR75-1	RPMI-1640 + 1% glutamine + 1% penicillin/streptomycin + 10% FBS
Breast cancer	T47D	RPMI-1640 + 1% glutamine + 1% penicillin/streptomycin + 10% FBS
Breast cancer	HCC-1428	RPMI-1640 + 1% glutamine + 1% penicillin/streptomycin + 10% FBS
Breast cancer	HCC-1187	RPMI-1640 + 1% glutamine + 1% penicillin/streptomycin + 10% FBS
Breast cancer	HCC-38	RPMI-1640 + 1% glutamine + 1% penicillin/streptomycin + 10% FBS + 1.5 g/L sodium bicarbonate + 10 mM HEPES + 1 mM sodium pyruvate
Breast cancer	Hs578T	DMEM + 1% glutamine + 1% penicillin/streptomycin + 10% FBS
Breast cancer	HCC-1954	RPMI-1640 + 1% glutamine + 1% penicillin/streptomycin + 10% FBS
Breast cancer	BT-474	RPMI-1640 + 1% glutamine + 1.5g/l sodium bicarbonate + 1% penicillin/streptomycin + 10% FBS
Breast cancer	BT-549	RPMI-1640 + 1% glutamine + 1% penicillin/streptomycin + 10% FBS + 1.5 g/L sodium bicarbonate + 10 mM HEPES + 1 mM sodium pyruvate
Breast cancer	HCC-1143	RPMI-1640 + 1% glutamine + 1% penicillin/streptomycin + 10% FBS + 1.5 g/L sodium bicarbonate + 10 mM HEPES + 1 mM sodium pyruvate
Breast cancer	HCC-70	RPMI-1640 + 1% glutamine + 1% penicillin/streptomycin + 10% FBS + 1.5 g/L sodium bicarbonate + 10 mM HEPES + 1 mM sodium pyruvate
Breast cancer	MDA-MB-436	L15 + 1% glutamine + 1% penicillin/streptomycin + 10% FBS without CO_2
Breast cancer	MDA-MB-231	DMEM + 1% glutamine + 1% penicillin/streptomycin + 10% FBS
Breast cancer	HCC-1937	RPMI-1640 + 1% glutamine + 1% penicillin/streptomycin + 10% FBS + 1.5 g/L sodium bicarbonate + 10 mM HEPES + 1 mM sodium pyruvate
Melanoma	A2058	MEM (Eagle) + 1% glutamine + 1% penicillin/streptomycin + 10% FBS
Melanoma	A375	DMEM + 1% glutamine + 1% penicillin/streptomycin + 10% FBS
Melanoma	MEL888	DMEM + 1% glutamine + 1% penicillin/streptomycin + 10% FBS
Melanoma	MEL624	DMEM + 1% glutamine + 1% penicillin/streptomycin + 10% FBS
Lung cancer	H23	RPMI-1640 + 1% glutamine + 1% penicillin/streptomycin + 10% FBS
Lung cancer	H522	RPMI-1640 + 1% glutamine + 1% penicillin/streptomycin + 10% FBS
Lung cancer	HOP-92	RPMI-1640 + 1% glutamine + 1% penicillin/streptomycin + 10% FBS
Lung cancer	HOP-62	RPMI-1640 + 1% glutamine + 1% penicillin/streptomycin + 10% FBS
Ovarian cancer	IGROV-1	RPMI-1640 + 1% glutamine + 1% penicillin/streptomycin + 10% FBS
Ovarian cancer	SKOV-3	RPMI-1640 + 1% glutamine + 1% penicillin/streptomycin + 10% FBS
Nonmalignant cell lines	MRC-5	MEM(Eagle) +1% glutamine + 1% penicillin/streptomycin + 10% FBS + 0.1 mM non-essential amino acids
Nonmalignant cell lines	HEK293T	DMEM + 1% glutamine + 1% penicillin/streptomycin + 10% FBS
Nonmalignant cell lines	GM08505	DMEM + 1% glutamine + 1% penicillin/streptomycin + 10% FBS
Nonmalignant cell lines	MCF-10A	DMEM/F12 + 5% horse serum + 20 ng/mL EGF + 100 ng/mL cholera toxin + 0.01 mg/mL insulin + 500 ng/mL hydrocortisone
Nonmalignant cell lines	MCF-12A	DMEM/F12 + 5% horse serum + 20 ng/mL EGF + 100 ng/mL cholera toxin + 0.01 mg/mL insulin + 500 ng/mL hydrocortisone

Supplementary Material 2. Primer sequences for RT-qPCR

qPCR primers		Sequence 5' to 3'	Reference
MAPT	F	GCTCATTAGGCAACATCCATC	Bou Samra et al. [2]
	R	GTCAGCTTGTGGGTTTCAATC	
CDA	F	CCCTACAGTCACTTTCCTG	Chabosseau et al. [3]
	R	CGGGTAGCAGGCATTTTCTA	
B2M	F	CGCTCCGTGGCCTTAGC	Kellouche et al. [4]
	R	GAGTACGCTGGATAGCCTCCA	
TBP	F	TGCACAGGAGCCAAGAGTGAA	Pasmant et al. [5]
	R	CACATCACAGCTCCCCACCA	
GAPDH	F	GAAATCCCATCACCATCTTCCAGG	West et al. [6]
	R	GAGCCCCAGCCTTCTCCATG	

References

1. Mameri H, Bieche I, Meseure D, Marangoni E, Buhagiar-Labarchede G, Nicolas A, et al. Cytidine Deaminase Deficiency Reveals New Therapeutic Opportunities against Cancer. Clin Cancer Res. 2017;23(8):2116-26.

2. Bou Samra E, Buhagiar-Labarchede G, Machon C, Guitton J, Onclercq-Delic R, Green MR, et al. A role for Tau protein in maintaining ribosomal DNA stability and cytidine deaminase-deficient cell survival. Nat Commun. 2017;8(1):693.

3. Chabosseau P, Buhagiar-Labarchede G, Onclercq-Delic R, Lambert S, Debatisse M, Brison O, et al. Pyrimidine pool imbalance induced by BLM helicase deficiency contributes to genetic instability in Bloom syndrome. Nat Commun. 2011;2:368.

4. Kellouche S, Mourah S, Bonnefoy A, Schoevaert D, Podgorniak MP, Calvo F, et al. Platelets, thrombospondin-1 and human dermal fibroblasts cooperate for stimulation of endothelial cell tubulogenesis through VEGF and PAI-1 regulation. Exp Cell Res. 2007;313(3):486-99.

5. Pasmant E, Masliah-Planchon J, Levy P, Laurendeau I, Ortonne N, Parfait B, et al. Identification of genes potentially involved in the increased risk of malignancy in NF1-microdeleted patients. Mol Med. 2011;17(1-2):79-87.

6. West AB, Kapatos G, O'Farrell C, Gonzalez-de-Chavez F, Chiu K, Farrer MJ, et al. N-myc regulates parkin expression. J Biol Chem. 2004;279(28):28896-902.
